# Statistically strong label-free quantitative identification of native fluorophores in a biological sample

**DOI:** 10.1038/s41598-017-15952-y

**Published:** 2017-11-17

**Authors:** Saabah B. Mahbub, Martin Plöschner, Martin E. Gosnell, Ayad G. Anwer, Ewa M. Goldys

**Affiliations:** 1 ARC Centre of Excellence for Nanoscale Biophotonics, Macquarie University, North Ryde, 2109 NSW Australia; 2 Quantitative Pty Ltd, ABN 17165684186, 116-118 Great Western Highway, Mt. Victoria, NSW 2786 Australia

## Abstract

Bioimaging using endogenous cell fluorescence, without any external biomarkers makes it possible to explore cells and tissues in their original native state, also *in vivo*. In order to be informative, this label-free method requires careful multispectral or hyperspectral recording of autofluorescence images followed by unsupervised extraction (unmixing) of biochemical signatures. The unmixing is difficult due to the scarcity of biochemically pure regions in cells and also because autofluorescence is weak compared with signals from labelled cells, typically leading to low signal to noise ratio. Here, we solve the problem of unsupervised hyperspectral unmixing of cellular autofluorescence by introducing the Robust Dependent Component Analysis (RoDECA). This approach provides sophisticated and statistically robust quantitative biochemical analysis of cellular autofluorescence images. We validate our method on artificial images, where the addition of varying known level of noise has allowed us to quantify the accuracy of our RoDECA analysis in a way that can be applied to real biological datasets. The same unsupervised statistical minimisation is then applied to imaging of mouse retinal photoreceptor cells where we establish the identity of key endogenous fluorophores (free NADH, FAD and lipofuscin) and derive the corresponding molecular abundance maps. The pre-processing methodology of image datasets is also presented, which is essential for the spectral unmixing analysis, but mostly overlooked in the previous studies.

## Introduction

Fluorescence microscopy with its various modalities is a powerful tool for studying biological processes in cells and tissues^1^. In order to reveal vital information about the imaged specimen, targeted labelling of multiple structures with extraneous fluorophores is usually performed. However, there are many applications where a more subtle, non-invasive approach might be desired, for example in the studies of fragile early embryos^2^. Fortunately, many chemical compounds in biological specimens, including nicotinamide adenine dinucleotide (NADH), nicotinamide adenine dinucleotide phosphate (NADPH), flavin adenine dinucleotide (FAD), flavin mononucleotide (FMN), retinol, cytochrome complex (Cyt. c), collagen, elastin and many other metabolites, enzymes, co-factors and vitamins in cells naturally exhibit fluorescence^2^. Importantly, the same compounds play a vital role in cellular metabolism^2^ and can therefore, provide highly informative fluorescent signatures^3^. Revealing the spatial distribution of cellular fluorophores and quantifying their concentration is therefore of utmost importance for the monitoring of biological processes^4–6^.

Native fluorescence in cells comprises a mixture of broad featureless spectra^7^ from multiple distinct molecular species. Each location in an imaged sample has a somewhat different chemical composition. This spatially-varying biochemistry is reflected in the spectral diversity of native fluorescence at various locations in the image. In this case, hyperspectral imaging and unsupervised linear unmixing make it possible to discriminate individual distinct fluorophores despite their highly overlapping spectra^8^, also in the presence of unavoidable image noise.

A range of approaches to unsupervised unmixing has been previously developed, mostly in the area of remote sensing^9–11^. The most popular unmixing methods such as N-FINDR^12,13^ require the presence of “pure” pixels that are the locations where only a single endmember is present. When pixel purity cannot be guaranteed, such as in our case of autofluorescence from biological samples, or in the presence of noise, alternative numerical analysis methods must be used, such as Minimum-Volume Transform (MVT)^14^, Sparsity Promoting ICE (SPICE)^15^, Minimum-Volume Enclosing Simplex (MVES) algorithms^16^, Minimum-Volume Simplex (MVSA)^17^, Dirichlet mixture Unmixing via Split Augmented Lagrangian (DUSAL)^18^ and Simplex Identification by variable Splitting and Augmented Lagrangian tools (SISAL)^19^. However, none of them except MVT^2,3^ have been previously applied to cell autofluorescence. They have only been applied to reflectance, where absolute reference points (such as in our case the fluorescence spectra of pure compounds) are not applicable. Moreover, the reflectance spectra must be less than unity, while fluorescence spectra are not similarly constrained; this is because fluorescence intensity can take arbitrary values. Furthermore, autofluorescence is usually more strongly affected by background noise than reflectance, with lower signal to noise ratio (SNR). In comparison, in conventional fluorescently labelled microscopy images of cells and tissues a typical range of SNR is 20–40 dB^20^. However, in this study, the autofluorescent images of retina cells are characterised by a far lower SNR of ~5 dB; similar SNR values were observed in previous investigations^2,3,21^.

The aim of this paper is to develop a state-of-the-art method to unmix cell autofluorescence. Here, unmixing with statistical minimisation^18^ is applied to biological hyperspectral fluorescence images for the first time. Earlier work accomplished the unmixing of cell autofluorescence by the MVT method^2,3^. Our approach of RODECA is based on DECA and DUSAL^18,22^ (Section 3.4) with appropriate modifications namely introducing SISAL and normalization of the spectra (see Supplementary Material, Section 4). By using artificial data with varying degree of known signal noise we estimate the unmixing accuracy (uncertainty of fluorophore spectra and their abundances), which was impossible in our previous approach^2^. We then employ this technique to unmix real biological autofluorescence images, we separate cellular autofluorescence into individual biochemical constituents and provide their cellular abundance maps.

### Background of hyperspectral unmixing

In hyperspectral imaging, two-dimensional images (*N* pixels each; here *N* ≈ 10^6^) of the same sample are captured in *L* different spectral channels (*L* = 18 in our study). The recorded fluorescence signals form the hyperspectral dataset described by a matrix ${\boldsymbol{y}}=[\,{y}_{ki}]$, where ${y}_{ki}$ is the pixel value in channel *k*, *(k* = 1, .., *L)* of image pixel *i*, (*i* = 1, *…*, *N)* ^23^. This matrix can be considered as a sum of the noiseless signal matrix ***x*** and the matrix of image noise ***n***, where ${\boldsymbol{y}}={\boldsymbol{x}}+{\boldsymbol{n}}$. Further analysis is based on a linear mixing model (LMM) which postulates that the observed spectrum in each pixel is a linear combination of a small number *p* of component spectra called “endmembers” with respective weights (that is concentration of the spectrum component) called “abundance” fractions^23–26^. For the unmixing to be accurate, the number of components needs to be smaller than the number of channels (*p* < *L*) Algebraically, in the LMM, the noiseless signals ***x***are expressed with the aid of an endmember matrix ${\boldsymbol{M}}=[{M}_{kj}]\,,$ (where, *k* = 1, *…*, *L*, *j* = 1, *…*, *p*) is weighted with abundance fractions specified in the abundance matrix ${\boldsymbol{s}}=[{s}_{ji}]$, (*j* = 1, *…*, *p*, *i* = 1, *…*, *N*) according to

1 $${\boldsymbol{x}}=[{x}_{ki}]=\sum _{j=1}^{p}{M}_{kj}{s}_{ji}={\boldsymbol{Ms}}.$$

Due to physical considerations, the abundance fractions in each pixel must be positive (${s}_{ji}\ge 0)$ and they must add to unity $\,\sum _{j=1}^{p}\,{s}_{ji}=1\,$ ^18,27^. The abundance matrix ***s*** contains the abundance fractions of each endmember for all *N* pixels and this makes it possible to easily construct the abundance map for the imaged sample. The *L*-dimensional vectors $\overrightarrow{{y}_{{i}}}$ forming columns of the matrix ***y***, represent the pixel spectra, while the *L*-dimensional vectors $\overrightarrow{{M}_{j}}$ forming columns of matrix ***M*** represent the endmember spectra. Equation (1) can be expressed as

2 $$\overrightarrow{{y}_{i}}=\overrightarrow{{M}_{1}}{s}_{1i}+\ldots +\overrightarrow{{M}_{p}}\,{s}_{pi}$$

This formulation makes it possible to see that the Equation (2) describes a convex set in *L*-dimensions span by the vectors $\overrightarrow{\,{M}_{j}}$. Therefore the pixel spectral vectors from the matrix ***x*** form a convex hull (a simplex) whose vertices are determined by the endmember spectral vectors^28^. There is *p* such vectors, so the convex hull has *p* vertices. The presence of noise ***n*** creates a fuzzy boundary of this convex hull.

In the unsupervised unmixing approach both matrices ***M*** and ***s*** are unknown, and, additionally, we do not know the number of endmembers ***p***. As previously indicated, the observed pixel signals in the hyperspectral dataset ***y*** are affected by the sensor noise coming from the image sensor and there may be other experimental sources of errors which are impossible to eliminate. Equation (2) then becomes:

3 $${\boldsymbol{y}}=\,{\boldsymbol{Ms}}+{\boldsymbol{n}},$$

The noise matrix ***n*** is also unknown. The procedure of unsupervised unmixing aims to establish the number of component spectra of a linear mixture and their identity, specifically to estimate optimal *p*, ***M*** and ***s*** none of which are known *a-priori*. Such statistical optimisation can give accurate results because hyperspectral imaging generates large datasets, so a large number *N* of experimental pixel spectra are available for analysis.

## Methods

### Hyperspectral Hardware Setup

We used a fluorescence microscope (Olympus iX71™) with a 40× water U12™ series objective, with the wide transmission in UV range. Selected bands of excitation wavelengths (centred at 334, 365, 375, 385, 395, 405, 415, 425, 435, 455, 475, 495 nm, each about 10 nm wide) are used to excite cell autofluorescence. Three epifluorescence filter cubes (details are given in Supplementary Material, Section 5) are available to measure single photon-excited emission of biological samples. With these 12 excitation sources and 3 filters, we created a total of 18 specific channels. Optical powers at the objective range from 0.01 µW (at 495 nm excitation with 587 nm emission, channel 15) to 42.8 µW (at 475 nm excitation with 587 nm emission, channel 14). The excitation sources are coupled by an optical fibre bundle with a 5 mm fused silica hexagonal homogenizer^29^. The excitation sources produce a reasonably flat approximately Gaussian distribution of illumination over the sample plane, whose flatness is further corrected digitally. All images are captured by Andor iXON™ camera (EMCCD, iXON 885 DU, Andor Technology Ltd., UK) operated below −65 °C to reduce sensor-induced noise. Some of the underpinning noise mechanisms depend on illumination level and they cannot be reduced by sensor cooling. The sensor size is 1002 × 1004 pixels.

### Data pre-processing

At the beginning of each experiment, reference images including calibration, water, and dark images are taken using our hyperspectral microscope system. These reference images are then used to pre-process the sample images. The pre-processing steps include image equalization, primary denoising with removing undetectable pixels and outliers (spikes or dips), background illumination flattening and cell segmentation. All this is carried out without changing the mathematical structure of the dataset. The pixel identification (image number, pixel coordinates, spectral channel etc.) are separately retained for the reconstruction of two-dimensional fluorophore abundance maps. The following pre-processing steps are carried out:

#### Image equalisation

In the image equalisation procedure, the intensity count at every channel is converted into the units of photons per pixel per second (PPS). This calculation helps to standardize images taken with different acquisition parameters, most notably electron-multiplication (EM) gain, and acquisition time. For our Andor iXON™ camera, the sample signal expressed in terms of photon per second,$\,{y}_{raw[PPS]}$, is given by:

4 $${y}_{raw[PPS]}(k,i)=\frac{({y}_{raw[digital]}(k,i)-B{O}_{[digital]})\times se}{{G}_{EM}\times QE\times {t}_{exp}}\,$$

here ${y}_{k,i[digital]}$ denotes the measured digital counts (in the range 0–2^14^). The bias offset $(B{O}_{[digital]})$ used in our setup is 100 counts. The camera sensitivity (*se*) for our readout rate of 13 MHz is 0.89. The EM gain (${G}_{EM}$) and exposure time (${t}_{exp}$) are adjusted by the operator taking into account the sample signals and they are generally different in different channels^30^. The quantum efficiency ($QE$) of camera sensor is also different for different channels (see Supplementary Material, Section 5).

#### Removing undetectable pixels

Two sets of dark images (acquired, respectively, with the microscope shutter open and closed) are taken to remove the undetectable pixels. Such undetectable pixels could be due to light blockages (e.g. by dust) between the sensor and the sample plane, or inactive camera pixels. The average of these two dark images is subtracted from all sample, water and calibration images, to correct for any pixels that are unresponsive.

#### Removing outliers (spikes)

Abnormal behaviour of sensor pixels in combination with high EM sensor gain may cause random sharp spikes or sharp dips in the image. To remove these outliers, a ‘threshold limiting window’ is scanned over all the images to locate these spikes or dips^31,32^. Then these specific data points are replaced with the values interpolated from immediately adjacent nine pixels^31,33^.

#### Image smoothing

The main sources of noise from EMCCD camera include illumination independent and illumination-dependent noise^34^. The illumination independent noises (e.g. dark-current shot noise, readout noise etc.) are minimised by using low sensor temperature (below −65 °C). Illumination-dependent noise (e.g. photon shot noise, clock induced charge noise, EM gain register noise etc.) are considered as multiplicative temporal and spatial noise^35^. The overall noise in autofluorescence images is a combination of illumination dependent noise which is approximately Poissonian, while the noise from the illumination independent sources can be modelled as a Gaussian noise^36^. We used Gaussian noise in our simulation as a proxy for the overall noise, because the Poisson’s noise amplitude cannot be modified independently from the signal. Here, we used the customised wavelet filter to remove the image noise for smoothing^2,29^ which facilitate improved capture of spectral information from a signal compared with standard frequency spectra produced by Fourier analysis^2^. The wavelets help to divide the signal into different scale components^37^ and thus these customized wavelet filters have proved to be a computationally efficient method of capturing textural information from filters or banks of filters with attractive attributes with potentially lossless coverage of the frequency spectrum^2^.

#### Removing background autofluorescence

The images are also affected by the unavoidable autofluorescence signals from the microscope slide, Petri dishes, dirt on sensors etc. These signals make additive contributions to all images. To remove these contributions we take two hyperspectral images of water in the petri dish used for imaging^38^. The smoothed average of these two images is denoted by $B(k,i)$. This smoothed average image, different for each channel, is subtracted from each sample image in this specific channel. See Section 3.2.7 for the description of smoothing.

#### Measurements of reference images and reference spectra

The microscope system is calibrated by taking hyperspectral images of a “calibration fluid” which in our experiments is a mixture of 30 μM NADH (quantum yield 0.019) and 5 μM riboflavin (quantum yield 0.24). Its composition has been adjusted so that the spectrum of the calibration fluid has non-zero response across all our spectral channels. The smoothed image of the calibration fluid is denoted by ${C}_{raw}(k,\,i)$. The excitation and emission spectra of the calibration fluid are also separately measured with fluorimeter, in our case a Cary Eclipse Fluorescence Spectrophotometer™, making sure these spectra span each channel used in hyperspectral image acquisition. The normalized spectrum,$\,f(k)$ of the calibration fluid thus obtained is used to correlate the hyperspectral images with the fluorescence spectra measured on a fluorimeter. This is necessary to be able to correctly assign the unmixed fluorophores, by using the spectra of pure reference fluorophores measured using the same fluorimeter with the same settings, in particular the same grating as used with the calibration fluid.

#### Relating the hyperspectral images to fluorescence spectra, image flattening and final image smoothing

Finally, the raw sample image,$\,{y}_{raw}(k,\,i),$ is corrected by using the averaged and smoothed background image $\,B(k,\,i)\,\,$and multiplied by the normalized spectrum of the calibration fluid,$\,f(k)$. Furthermore, the smoothed image of the calibration fluid is used to correct for the somewhat uneven (approximately Gaussian) illumination of the field of view^39^. This is done by dividing the sample image in each channel (after subtracting of the smoothed water image) by the relevant smoothed image of the calibration fluid^2^. These corrections are specified in Equation 5:

5 $$\,y(k,\,i)=\frac{f(k)\times ({y}_{raw}(k,i)-B(k,i))}{({C}_{raw}(k,\,i)-B(k,\,i))}$$

#### Cell segmentation

In order to calculate cellular features, cellular images need to be segmented into individual cells. In this work, retina cells were selected manually with the overlaid DIC image. The normalized autofluorescence intensity in each cell is documented in the $\,{y}_{ki}$ matrix. The image number, pixel coordinates *i* and the spectral channel indices *k* are saved separately for the reconstruction of two-dimensional fluorophore abundance maps.

### Identifying the optimised vector subspace by HySime and data decorrelation

**Figure 1:**
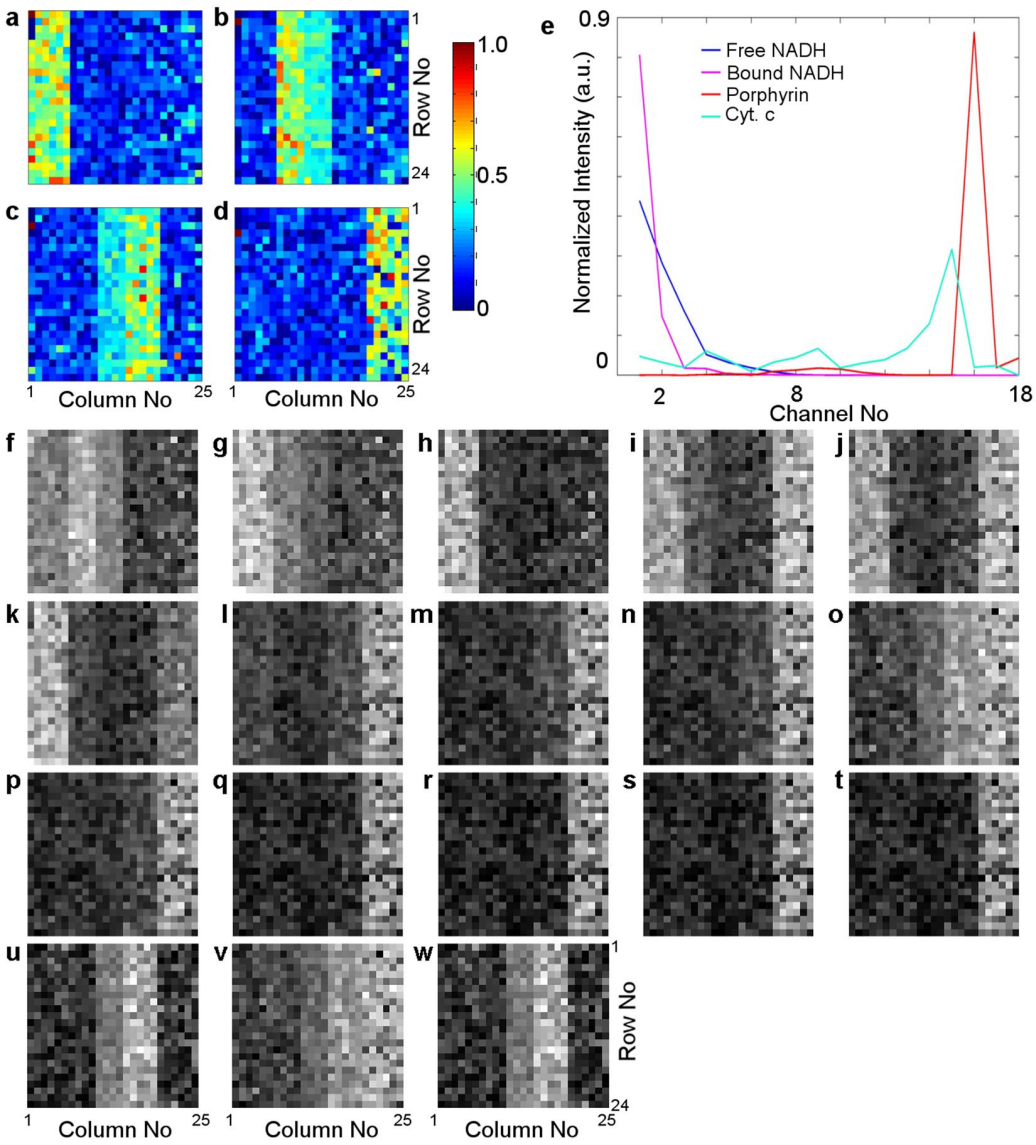
(**a**–**d**) The artificial abundance map ${{\rm{s}}}_{[4\times 600]}$ of the total of 600 (24 × 25) pixels containing the fractions of each of four fluorophores (**a**) free NADH, (**b**) bound NADH, (**c**) porphyrin and (**d**) Cyt. c. This abundance map corresponds to case D. (**e**) The normalized reference spectra are shown for free NADH (blue), bound NADH (magenta), Cyt. c (cyan) and porphyrin (red). (**f**–**w**) Simulated hyperspectral image set (${\boldsymbol{y}}={\boldsymbol{Ms}}+{\boldsymbol{n}}$) assuming zero noise (${\boldsymbol{n}}=0$) taken in $L=18$ different spectral channels. The corresponding channel numbers are given in Supplementary Material, Section 5. Images (**f**–**w**) are rescaled for better visualization as a grey colour-map, where the minimum value is displayed as black, and the maximum value (varying at each channel) is displayed as white (details of the colour ranges are given in Supplementary Table 2).

Hyperspectral datasets pose two main problems for the analysis. Firstly, the number of channels *L* may be large and this generates high volume of data which then leads to computational complexity. Secondly, hyperspectral datasets are highly correlated. This is apparent in the images in spectrally close channels which look closely similar (as illustrated in Fig. 1f–w). In this work, we address these two problems of dimensionality reduction and decorrelation by a standard approach called HySime^17,28^. In HySime we first calculate the ($L\times L$) signal covariance matrix which is then diagonalised. In the next step, all possible subsets of its orthogonal eigenvectors are individually examined. A numerical criterion derived in Supplementary Material (Section 1, Equation 14) is then evaluated for each of these subsets. This criterion uses the data matrix ***y*** only, and it minimises the mean square error between the unknown noiseless signal matrix ***x***, and the noisy observed signal ${\bf{y}}={\boldsymbol{x}}+{\boldsymbol{n}}$. The subset which best satisfies this criterion is then selected as the set of *p* basis vectors (${{\boldsymbol{E}}}_{1,\mathrm{.}.,p})$ spanning the optimised “signal” subspace (details are presented in Supplementary Material Section 1.3). The number of these new basis vectors is generally less than *L*. The original data vectors ***y*** (pixel spectra) are then orthogonally projected onto this optimised subspace. The resulting new *p*-dimensional data vectors form an optimal low-dimensional representation of the original data. These data vectors provide the input to the DECA procedure.

### DECA procedure and its modifications

In this work, the Robust Dependent Component Analysis (RoDECA) algorithm is used for extracting the native fluorophores and their corresponding abundance matrix which is a modified version of DECA^18^. DECA is an unsupervised hyperspectral unmixing method based on the assumption of the linear mixing model. DECA is well suited to highly mixed datasets, where there are no pure pixels present. In the DECA analysis, the intermediate *p* × *p* endmember matrix ***A*** is initially estimated blindly, by simulating the abundance matrix ***s*** as a mixture of Dirichlet densities^18^ (details are given in Supplementary Material, Section 3). The main advantage of using the Dirichlet densities is that this approach automatically enforces the constraints of non-negativity and unity sum, and thus it accounts for statistical dependence usually found in hyperspectral data which satisfy the LMM conditions. The Dirichlet densities are also well suited to model complex distributions in which the ‘mass probability function^18^, is scattered over a number of k-clusters inside the simplex^40^. Defining ${\boldsymbol{W}}\equiv \,{{\boldsymbol{A}}}^{-1}$ we then obtain that the abundance map ***s*** in the subspace is given by:

6 $${\boldsymbol{s}}={\boldsymbol{Wx}}.$$

The maximum likelihood estimation of ***W*** ^41,42^, is carried out using an iterative expectation maximization (EM) algorithm^18^. This algorithm iterates between the “expectation” step and “maximisation” step^43^ following the General Expectation Maximisation (GEM) approach^28,43^ (details are given in Supplementary Material, Section 3.3 and Section 3.4). The optimization problem in the case of biological autofluorescence is very challenging owing to the nonconvex term $\,\mathrm{log}\,|\det ({\boldsymbol{W}})|$, originally introduced by the DUSAL function in DECA and that is why in this work we put forward a more relaxed optimization process.

In our RoDECA analysis^44^, a soft negative function is introduced to determine the $\mathrm{log}\,|\det ({\boldsymbol{W}})|$by simplex identification via the split augmented Lagrangian method (SISAL)^19^ (details are given in Supplementary Material, Section 4.1, Equation 38). SISAL optimises the hard nonconvex problem (where there is no sharp vertices) by using a soft negative function^19^ for the non-smooth convex hull^45,46^. The SISAL method was chosen because it helps to maintain the full rank with account of physical constraints (non-negativity and sum-to-one), in which the pure pixel assumption is violated^19^. Normalization has been introduced to the extracted endmember matrix ***M*** (after SISAL), ensuring the non-negativity and normalized values at each channel (details are given in Supplementary Material, Section 4.2). When the input data has been normalized, the extracted data should be normalised as well to prevent undesired discrimination of the same biochemical with different intensities^47^, which cannot achieved by the SISAL or DUSAL alone.

### Cell preparation and imaging conditions

In this study, we used the RGC-5 cell line, a neuronal cell line of retinal origin^48^ derived from the mouse retinal photoreceptor cells^49^. The cells were maintained in Dulbecco’s Modified Eagle’s medium (DMEM-high glucose, Sigma Aldrich, D5796) containing 10% bovine serum (Sigma Aldrich, 12133 C) at 37 °C with 5% CO_2_ incubator. Approximately, 2.0 × 10^5^ cells were seeded in each culture dish 12 hours before imaging.

The RGC-5 cells were cultured into three 35 mm plastic culture dishes with 18 mm diameter wells and # 1.5 gridded coverslip bottoms (Cell E&G, USA, and # GDB0004-200) for the preparation of hyperspectral imaging. Then DMEM medium containing 10% fetal bovine serum was removed (as it has own autofluorescence) and the cells were washed with Dulbecco’s phosphate-buffered saline (DPBS, Sigma Aldrich, D8537) three times. After that 1 ml of non-fluorescent Hanks Balanced Salt Solution (HBSS, Life Tech, Australia, 14025076), was added to each dish for imaging. During the imaging session, the temperature was maintained at 37 °C. The cells located over a reference grid at the bottom of the dishes were imaged using 18 fluorescence channels. These gridded coverslips are also useful for further correlation of hyperspectral imaging with labelled imaging, not reported here. A single wavelength image is typically acquired for between 0.5–15 seconds depending on the sample and wavelength, approximately 3 minutes for the entire stack of images for all 18 spectral channels.

## Results and Discussion

### Overview of Hyperspectral Unmixing Analysis

The hyperspectral unmixing analysis involves several steps. First, hyperspectral images are taken with modified autofluorescence microscope setup (Section 3.1); these form the hyperspectral dataset ***y***. It is desirable to unmix all images from a biological experiment together, as this enables their more accurate comparisons, but single images can also be unmixed. The hyperspectral dataset ***y*** then undergoes data pre-processing (Section 3.2). Subsequently, linear unmixing is carried out for the dataset ***y*** (Sections 3.3 and 3.4). It involves several steps, including; decorrelation and estimation of the number of endmembers *p* by the HySime method^28^, and estimation of the noise matrix ***n*** ^28,50^ in the least square error sense^50^ with an iterative statistical procedure (Section 3.3). This is followed by endmember extraction and generation of the abundance matrix with the RoDECA (Section 3.4). This step iteratively derives the abundance matrix from an initial mixture of Dirichlet densities and endmember spectra. The procedure is blind, and it does not require any geometrical algorithms^9–11,18^.

### Determining the accuracy of unmixing using simulated data

In this work, we first applied the RoDECA analysis to an artificial data set, in order to determine how accurately we can unmix fluorophores for varying amounts of deliberately added noise. Such artificial hyperspectral fluorescence data make it possible to generate noisy data with a varying signal to noise ratio. Mathematically, we generate a reasonably realistic “zero-noise” dataset ${\boldsymbol{x}}={\boldsymbol{Ms}}$ by selecting real biological fluorophores and using their real spectra. Then we generate a suite of different white Gaussian noise matrices ***n*** with different noise amplitudes and thus produce artificial signal matrices $\,{\boldsymbol{y}}={\boldsymbol{Ms}}+{\boldsymbol{n}}$. We then calculate the ***M*** and ***s*** by using our developed RoDECA procedure and compare them with original ***M*** and ***s*** for further analysis.

#### Preparation of artificial data sets

**Figure 2:**
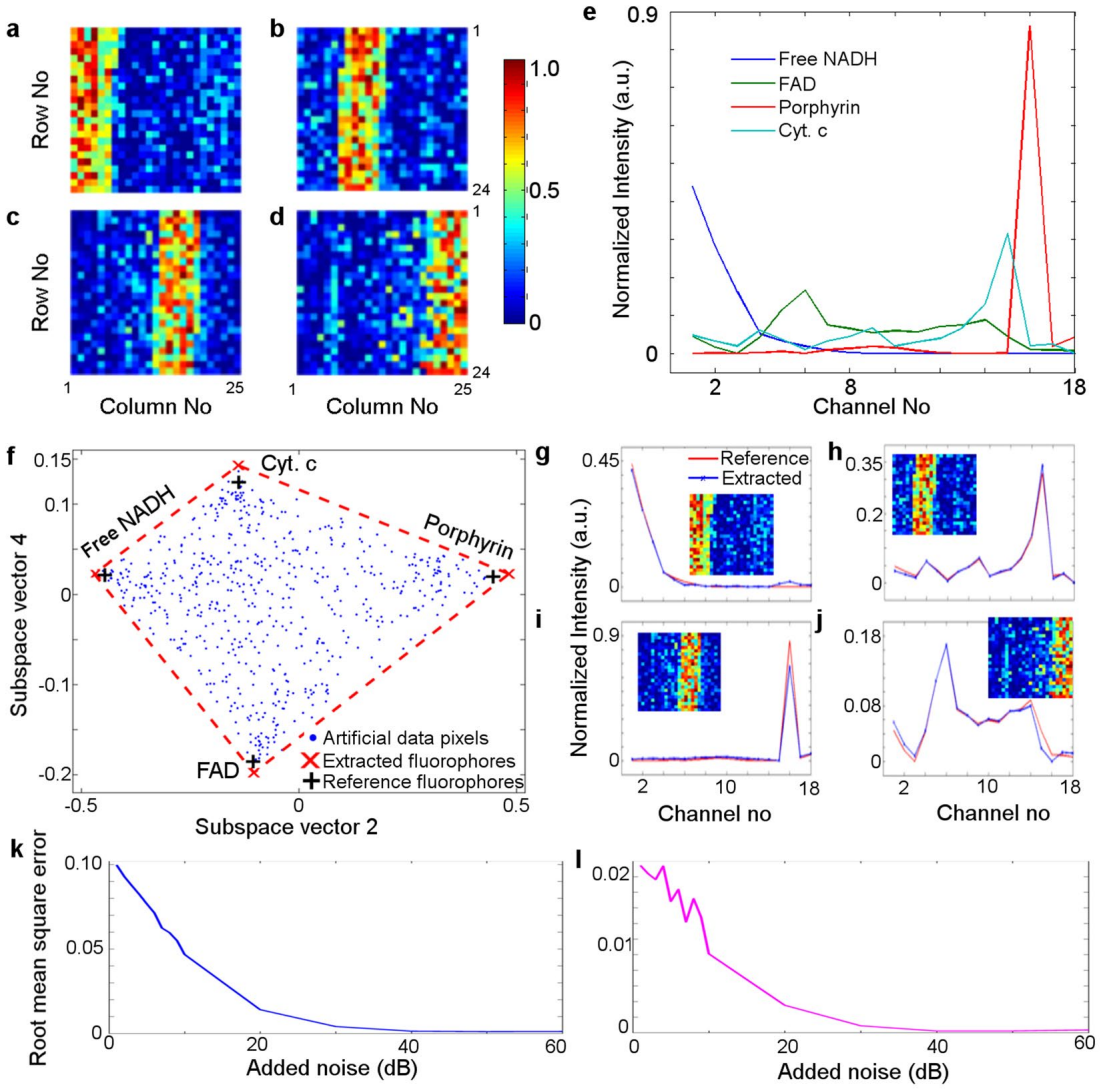
(**a**–**d**) The artificial abundance map ${{\rm{s}}}_{[4\times 600]}$ of the total of 600 (24 × 25) pixels containing the fractions of each of four fluorophore (**a**) free NADH, (b) FAD, (**c**) porphyrin and (**d**) Cyt. c. This abundance map corresponds to case A. (**e**) The normalized reference spectra are shown for free NADH (blue), FAD (green), Cyt. c (cyan) and porphyrin (red). (**f**) Simplex representation of simulated data corresponds to case A. The blue dots represent 600 data points projected onto the plane span by subspace eigenvectors 2 and 4, as in this plane all 4 vertices of the simplex were clearly visible. The red crosses represent the endmembers extracted by unsupervised unmixing using modified DECA with red enclosure marking the surface of the simplex and black crosses representing the reference spectra of free NADH, FAD, porphyrin and Cyt. c. (**g**–**j**) The endmembers spectra extracted using DECA (in blue) are compared with the reference spectra of (**g**) Free NADH, (**h**) FAD, (**i**) porphyrin and (**j**) Cyt. c (red). In Figures (**f**–**j**) the SNR is 20 dB. (**k**–**l**) Root mean square error calculations of the extracted (**k**) abundance matrices (in blue) and (**l**) endmember spectra (in magenta) for various levels of applied Gaussian noise ***n***.

We generated pre-determined abundance maps ***s***, by creating two-dimensional images $N=24\times 25\,\,$pixels representing maps of abundance fractions and assigned them to each of the four chosen fluorophores free NADH, bound NADH, porphyrin and cytochrome complex, Cyt. c (see example in Fig. 1a–d) and free NADH, FAD, porphyrin and cytochrome complex, Cyt. c (see example in Fig. 2a–d). These fluorophores are commonly found in biological samples, and they are related to cell metabolism. Among them, the ratio of NADH and FAD reflects the metabolic rate in cells^4,51^. The spectra of chosen fluorophores in 18 channels are shown in Fig. 1(e) and in Fig. 2(e). We emphasise that these spectra are neither emission nor excitation spectra, they are proportional to fluorescence intensities of the respective fluorophores in the spectral channels whose specifications are given in Supplementary Material, Section 5. The abundance map ***s*** satisfies physical constrains, namely that all four abundance matrices in each pixel add to unity and they are all positive.

The simulated dataset ***x*** with zero noise (${\boldsymbol{n}}=0$) has been obtained by multiplying the endmembers spectra ***M***, shown in Fig. 1(e), by the abundance matrix ***s***, the abundance matrix of case D shown in Fig. 1(a–d). The simulated data set is shown in Fig. 1(f–w), where we present images in all spectral channels with image scaling for better visualisation. During the image scaling process, the highest pixel value was assigned as white colour and the lowest pixel value was black (details of colour scales are given in Supplementary Table 2). The hyperspectral dataset cube with 18 channel images was then reshaped to form the ${y}_{ki}$ matrix used in the unmixing analysis. The pixel coordinates ($k,\,i$) were saved separately for further reconstruction of the abundance maps from the extracted $\,{{\boldsymbol{s}}}_{ji}$.

Four sets of artificial data (referred to as cases A, B, C, D) were generated with varying degree of spatial and spectral overlap. In case A, the maps of the abundance fraction have been designed with distinctive stripes for ease of visualisation. We assigned high abundance (up to 80–85%) to each of the four fluorophores in a separate region of the image. These regions do not overlap (Fig. 2a–d). The abundances in our artificial data are randomly generated to be able to represent a wide variety of abundance mixtures in the real data. This enables the reader to better visualise the convex hull surrounding the data points. This process is separate to the addition of the Gaussian image noise.

The remaining three datasets (B, C, D) are characterised by a higher degree of spatial overlap (between two fluorophores - Case B and three and four fluorophores - Case C). These results are presented in Section 6.2 and 6.3 in the Supplementary Material. The dataset with high spectral overlap (between free NADH and bound NADH - Case D) is presented in Fig. 1).

In case B, FAD and porphyrin spatially overlap in columns 11 through to 16 with the abundance fractions of around 50% each. The simplexes of the artificial data, as well as the spectra of extracted fluorophores compared with reference spectra in the presence of added Gaussian white noise with the SNR of 10 dB, 20 dB, 40 dB and 60 dB, are shown in Supplementary Figs 9, 10, 11 and 12 respectively.

In case C, FAD and porphyrin spatially overlap in column 11 through to 16 which produces the abundance fraction of around 50% for both FAD and porphyrin. Moreover, the regions of free NADH and Cyt. c spatially overlap in rows 11 through to 16 with the abundance fractions of around 50% each. RoDECA unmixing was carried out with added Gaussian white noise for the SNR of 10 dB, 20 dB, 40 dB and 60 dB. The results are shown in Supplementary Figs 14, 15, 16 and 17 respectively.

In Case D, we chose two spectrally similar fluorophores, free NADH and bound NADH (Fig. 1e). Moreover, in this example, we introduced significant spatial overlap between bound NADH and porphyrin, in column 11 through to 16, with abundance fractions of around 50% each. In order to establish the robustness of RoDECA unmixing, the unmixing analysis was carried over with a broad range of added Gaussian white noise with SNR from 10 dB to 60 dB. The simplexes of the artificial data, as well as the spectra of extracted fluorophores compared with reference spectra for the SNR of 10 dB, 20 dB, 40 dB and 60 dB, are shown in Supplementary Figs 18, 19, 20 and 21 respectively.

#### DECA unmixing of the artificial data set

We first discuss the results of unmixing of the artificial dataset (Case A) ${{\boldsymbol{y}}}_{[18\times 600]}$with added Gaussian white noise yielding the signal to noise (SNR) ratio of 20 dB. The dataset ***y*** is processed with HySime, to obtain the number of fluorophores (in this case *p* = 4), and the corresponding subspace vectors $\,{{\boldsymbol{E}}}_{1,\ldots ,p}$. In this subspace, the dimensionality of the input data ${{\boldsymbol{y}}}_{ki}$ is reduced, and the input data projected onto the subspace is now denoted ${{\boldsymbol{y}}}_{ji}$ (*j* = 1, *…*, *p*) (Section 3.3). Following the DECA analysis of this dataset, we obtain both ***M*** and ***s***, both these matrices also projected onto the same subspace.

Both abundance maps (in Fig. 2a–d) and corresponding spectra (in Fig. 2e) are presented for this study. For display purposes, the artificial dataset ${{\boldsymbol{y}}}_{ji}$ was further projected onto the signal subspace vectors 2 and 4, see Fig. 2(f) (details are given in Supplementary Material, Section 1.3 and Section 2). The channel signals (spectra) of reference fluorophores were similarly projected (black crosses). As expected, the data points (blue dots,$\,N=600$), form a convex simplex with four vertices. The vertices correspond to the extracted endmember spectra projected onto the chosen subspace. It can be seen that the endmembers and the original fluorophores closely coincide. Figure 2(g–j) shows the spectra of endmembers extracted by using DECA analysis compared with reference fluorophore spectra for the added noise of SNR 20 dB, and the corresponding abundance maps. As anticipated, for this high signal to noise ratio the unmixed endmembers and reference fluorophores are practically identical.

In order to establish the robustness of DECA unmixing, this analysis was extended to cover a broad range of added Gaussian white noise with SNR from 1 dB to 60 dB. The simplexes of the artificial data (Case A), as well as the spectra of extracted fluorophores compared with reference spectra for the SNR of 60 dB, 40 dB and 10 dB, are shown in Supplementary Figs 4, 5 and 6. The root mean square error of abundance $\,{\xi }_{s}$ is determined by using Equation 7 (see^18^). Here ${s}_{cal}(j,i)$ denotes the calculated abundance matrix and ${s}_{org}(j,i)$ is the original abundance matrix.

7 $${\xi }_{s}=\frac{1}{p}\sum _{j=1}^{p}\frac{1}{N}\sum _{i=1}^{N}\sqrt{{s}_{cal}(j,i)-{s}_{org}{(j,i)}^{2}}$$

The spectrum error ${\xi }_{M}$is calculated as per Equation 8. Here, ${M}_{cal}(k,j)$ and ${M}_{org}(k,j)$ represent the calculated and original matrices of endmember spectra, respectively.

8 $${\xi }_{M}=\frac{1}{p}\sum _{j=1}^{p}\frac{1}{N}\sum _{k=1}^{L}\sqrt{{M}_{cal}(k,j)-{M}_{org}{(k,j)}^{2}}$$

Figure 2(k,l) shows the root mean square error of the abundance fraction and for the endmember spectra for different levels of added Gaussian white noise. They confirm the expected trends such as lower errors at a lower value of added noise. As expected, at high SNR the individual maps of abundance fractions obtained from the RoDECA analysis and the original abundance maps are almost identical, with the abundance error for the SNR of 40 dB and 20 dB of 0.0014 and 0.0142 respectively. The spectrum error for the SNR of 40 dB and 20 dB is also very small with the respective values of 0.0002 and 0.0035.

Similar analysis was carried out using artificial data in cases B, C and D. The root mean square error of abundance $\,{\xi }_{s}$ for Cases B - D is presented in Supplementary Figure 22 (blue coloured cross, diamond and asterisk symbols). The root mean square error of spectrum $\,{\xi }_{M}$ for Case B - D is presented in Supplementary Figure 23 in red coloured cross, diamond and asterisk symbols.

We also performed an additional study to benchmark RoDECA with other well-established methods including SISAL^19^, VCA^52^, DUSAL^18^ and MVSA^17^. The results are shown in Supplementary Figure 24. The root mean square error of spectrum $\,{\xi }_{M}$ of RoDECA is the smallest (0.0165), while it is 0.0867, 0.0566, 0.0897 and 0.0667 for SISAL, VCA, MVSA, and DUSAL respectively.

### DECA unmixing in a biological experiment

**Figure 3:**
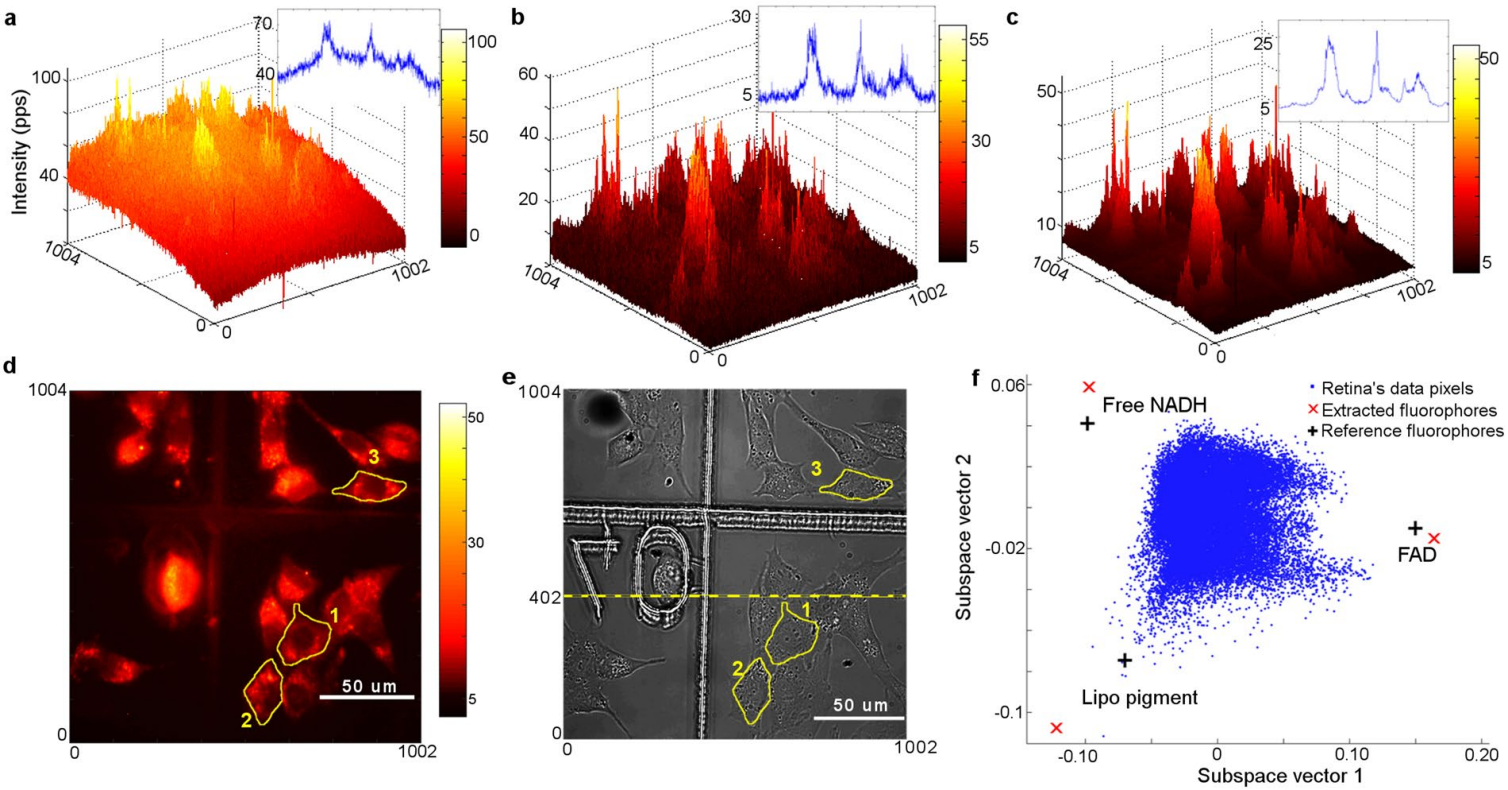
(**a**–**e**) Selected single channel image (1004 × 1002 pixels) of the retina cells in consecutive stages of pre-processing. (**a**) Raw data $(\,{{\boldsymbol{y}}}_{raw})$ of retina cells in Channel 5 before any image pre-processing. Intensity is given in PPS units, as explained in Section 3.2.1, (**b**) the same image after background subtraction (Section 3.2.5) and image flattening (Section 3.2.7) by using the image of the calibration fluid. Intensity is given in PPS units. (**c**) Smoothed image after wavelet smoothing (Section 3.2.4). The intensity scale is now normalized. (**d**) The intensity map for Fig. 3(c), where cells are now clearly distinguishable in the smoothed image. Three cells are outlined (yellow line) and numbered for individual cell analysis. (**e**) The corresponding differential interference contrast (DIC) image with the three cells outlined in Fig. 3(d). Note that the grid itself and the grid number seen in the DIC image is not observed in the fluorescence image. (**f**) The simplex representation of the retina data on projection on the subspace 1 and 2 of the signal subspace. Data pixels - blue dots, extracted endmembers - red crosses. The location of the reference spectra of free NADH, FAD and lipofuscin are marked with black crosses. The total of 209028 pixels have been analysed.

In our experiment, we collected the hyperspectral images of retina cells, and we also took a series of reference images of the calibration fluid and water as well as dark images. Figure 3(a) shows a raw single channel image (1004 × 1002 pixels) of the retina cells in channel 5 where the digital counts in the hyperspectral images were converted into equivalent units of photons per pixel per second (PPS). The images have then been pre-processed as described in the Methods section. The pre-processing steps included image equalization, removing undetectable pixels, removing background fluorescence and flattening the images. In this experiment higher EM gains and longer acquisition time with multiple averaging is used to optimise image quality. The average of two dark images is then applied to remove the undetectable pixels. The image is flattened with a normalized spectrum of the calibration fluid, shown in Fig. 3(b) and multiplied by the appropriate value from the spectrum of the calibration fluid. After that, a customized wavelet filter is used to smooth the raw image, as presented in Fig. 3(c). This procedure increases the SNR to 8.5 dB from the original value of 4.6 dB. The insets of Fig. 3(a,b,c) show the intensity value across the image (in blue) for the ease of comparison between these pre-processing steps. Figure 3(d) shows the intensity plot of the smoothed image of the retina cell, presented in Fig. 3(c), whereas Fig. 3(e) shows the DIC image for the same region. Finally, the image was segmented manually using the superimposed DIC, Channel 2 and Channel 5 images.

**Figure 4:**
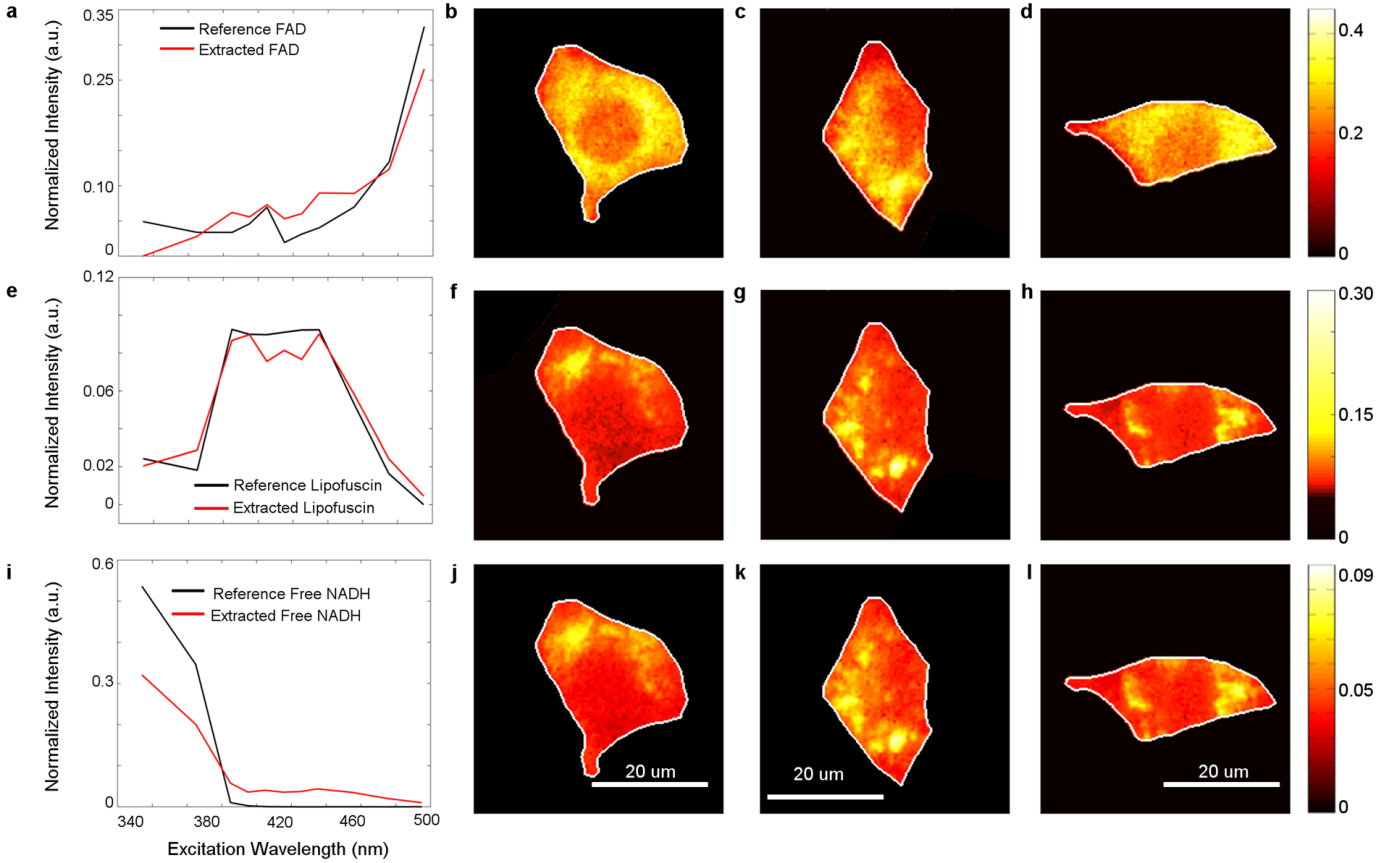
Abundance map representation of three retina cells, marked (as 1, 2, 3) in the DIC image in Fig. 3(e). First column (panels a,e,i) shows the extracted and reference spectra in red and black respectively. Figures (**b**,**f**,**j**) (**c**,**g**,**k**) and (**d**,**h**,**l**) represent the three abundance maps for the first, second and third cell, respectively. Here the first row shows the abundance map of FAD (**b**,**c** and **d**), the second row illustrates the abundance map of Lipofuscin (**f**,**g** and **h**) and third row presents the abundance map of Free NADH (**j**,**k** and **l**).

After pre-processing, the DECA analysis was used for the unmixing of this hyperspectral dataset. Figure 3(f) shows the simplex for the analysed dataset from the retinal cells investigated here. The extracted fluorophores are shown as red crosses, whereas the corresponding reference spectra are shown with black crosses. The vertices are not clearly pronounced, compared with the artificial data, which is indicative of highly mixed fluorophores. The analysis of such dataset using alternative minimum volume methods MVT, MVES or MVSA^2,3^ would be challenging due to a higher degree of associated complex noise and highly mixed fluorophores. Since the endmembers are highly mixed, i.e., there is no spectral vector near the vertices nor near facets, the minimum volume methods are unable to satisfy their underpinning assumption of at least p − 1 spectral vectors on each facet of the data simplex^18^. In this case, statistical methods such as DUSAL^18^ or our RoDECA are necessary to handle such highly mixed data sets. However, with RoDECA analysis, the three most abundant fluorophores have been successfully identified. Figure 4a,e,i compares the (excitation) spectra of the extracted and reference fluorophores which shows that the extracted endmembers are similar to FAD, NADH and lipofuscin. The root mean square error of spectrum error was measured 0.0275 with respect to all 16 channels and root mean square error of individual spectra for FAD, NADH and lipofuscin is 0.0234, 0.0484 and 0.0107 respectively, presented in Fig. 4a,e,i.

These assignments are in agreement with earlier works in retina and retinal cells which reported the presence of NAD(P)H, FAD, and retinol^53,54^, identified by the two photon microscopy^53,55^ or *ex vivo* fluorescence microscopy^56–58^, while lipofuscin, has been detected using single photon imaging methods^59–61^. Our RoDECA unmixing methodology makes it possible to identify NADH and FAD by single photon fluorescence in a statistically robust way. We note that lipofuscin in retinal cells is composed of many different fluorophores with a broad excitation and emission profile^62^, which gives a very rounded vertex in our simplex representation in Fig. 3(f).

The obtained abundance maps (Fig. 4b,c,d,f,g,h,j,k,l) show cell nuclei (dark ovals), confirming that we have captured aspects of cellular morphology. These abundance maps are expected to reflect relative proportions of fluorophore concentrations weighted with their fluorescence quantum yield. We note however, that some variations of the fluorescence quantum yield may be expected, as a result of the highly diversified chemical environments within a cell and pH variations. The FAD and NADH are found to be more pronounced in the perinuclear region of the cytosol. This is consistent with their mitochondrial localisation. Both FAD and NADH are the essential component of electron transport chain and used as an indication of mitochondrial function^63^ also in retina^64^, where they can serve as functional indicators of the ganglion cell layer^65–67^. The ratio of the fluorescence intensity of these fluorophores (NADH/FAD), the NADH redox ratio, is a marker of the metabolic state^68^. The third extracted fluorophore, lipofuscin is also to be expected in the retinal cells^69^, because these cells accumulate this by-product of phagocytosis^62,70,71^. The extracted abundance map of lipofuscin at Fig. 4(g) clearly show the lipofuscin granules. Lipofuscin granules are usually found in retinal pigment epithelial cells (RPE)^69^, which allows monitoring the health conditions of the retina cells^72^. The density of lipofuscin granules in RPE cells in mice increased with age^73^, and it is regarded as the key pathogenic indicator for age-related macular degeneration (AMD). As the eye is an ideal organ for non-invasive imaging, the presented methodology might contribute to improved quantification and evaluation of lipofuscin for clinical *in vivo* diagnostics^58,74^.

## Conclusions

This paper presents the mathematics and experimental application of the unsupervised unmixing analysis applied to biological autofluorescence images which contain significant levels of noise. Earlier hyperspectral imaging experiments analysed by unsupervised unmixing used mostly reflectance, however, reflectance is a significantly different modality from fluorescence due to the availability of the absolute reference spectra in fluorescence. Earlier studies of decomposition of multiply labelled fluorescence images of cells and tissues^75,76^ were limited to much higher SNR than in the current work, ranging from 20 dB to 40 dB^20^. The image pre-processing steps include the standardization of the measured fluorescence intensity counts. This is followed by the subtraction of background autofluorescence and image flattening. These help to reduce illumination independent artefacts. The illumination-dependent complex noise is treated by a customized wavelet filter^2^ for primary smoothing. The dataset is then decorrelated by HySime allowing the data dimensionality to be reduced. Subsequently, both unknown a-priori unknown endmembers and abundances are extracted with the RoDECA analysis, by introducing SISAL and the normalization of the spectra. Unmixing of artificial data has been conducted to quantify the noise-dependent unmixing error for both abundances and extracted spectra, which help evaluate the credibility of our RoDECA analysis. The real biological dataset of retina cells then has been tested to identify individual biochemical components of cellular autofluorescence and provide their relative abundance maps. These maps reflect the cellular morphology and correctly localise core cellular features. With its robust unmixing statistical analysis, this study reports a versatile unsupervised unmixing method for label-free quantitative identification of native fluorophores in a biological sample and it yields new insights into cytometry of retinal cells.

Quantitative analysis of autofluorescence features presented here has a vast array of potential applications in the analysis of cells and tissues, from an understanding of metabolic activity to developing future clinical tools for diagnostics and therapy monitoring^2^. In this context, it is especially important that spectral fluorescence microscopy is a relatively inexpensive imaging methodology. As native fluorescence is such a universal and informative biological characteristics, the hyperspectral unmixing technology of autofluorescence has a potential for impact on the field of life sciences and biomedicine.

## Electronic supplementary material

Supplementary Material
