# Supplementary material for: Statistically strong label-free quantitative identification of native fluorophores in a biological sample
Source: Sci Rep. 2017 Nov 17;7:15792. doi: 10.1038/s41598-017-15952-y (PMC5693869; doi:10.1038/s41598-017-15952-y)
Supplement: Supplementary file 1 — Supplementary Material [file 41598_2017_15952_MOESM1_ESM.pdf]

Supplementary Material
Statistically strong label-free quantitative identification of native fluorophores in a biological sample

Saabah B. Mahbub[1,*], Martin Plöschner[1], Martin E. Gosnell[1,2], Ayad G. Anwer[1], Ewa M. Goldys[1,*]

[1] ARC Centre of Excellence for Nanoscale Biophotonics, Macquarie University, North Ryde 2109, NSW Australia.

[2] Quantitative Pty Ltd, ABN 17165684186, 6 Townsend Circuit, Beaumont Hills NSW 2155, Australia.

[*] Corresponding author, E-mail address: saabah.mahbub@gmail.com, ewa.goldys@mq.edu.au

1 Subspace identification

In the main article, we have explained the process of collection and processing of raw spectra in each pixel of the image. The spectrum in a pixel denoted by i ($i \in 1 \ldots N$, where N is the number of pixels in the image) can be represented by a vector $\mathbf{y}^{(i)}$ in a space $\mathbb{R}^L$ (L is the number of spectral bands collected). The $\mathbb{R}^L$ space spans (covers) all the possible outcomes of the spectral measurements, however, in any realistic biological experiment only a very limited subspace of $\mathbb{R}^L$ is necessary to describe the spectral measurement fully and with minimal loss of information. The identification of the spectral subspace has several advantages, namely gains in computational time, reducing the complexity and, crucially, substantially improving the signal-to-noise ratio of the spectral measurements. In this section, we describe the unsupervised approach to subspace identification, that correctly identifies the subspace dimension, while, simultaneously, removing a significant portion of the noise from our spectral measurement.

Each observed spectral vector $\mathbf{y}^{(i)}$ is a sum of signal spectra $\mathbf{x}^{(i)}$ and additive noise $\mathbf{n}^{(i)}$ (from various sources):

$$\mathbf{y}^{(i)} = \mathbf{x}^{(i)} + \mathbf{n}^{(i)} \tag{1}$$

Our first goal is to find a subspace $\mathbb{R}^p \subset \mathbb{R}^L$, where $p < L$, so that the remaining coordinates p to L contain minimal error. Once we find such a subspace, we project the spectral vectors $\mathbf{y}^{(i)}$ onto it.

1.1 The directions of largest variance

Imagine a hypothetical situation in which all the observed spectral vectors $\mathbf{y}^{(i)}$ exhibit zero variance in one of the coordinates (spectral bands). Such constant coordinate is superfluous and can be removed. The trimmed spectral vectors are then part of subspace $\mathbb{R}^{L-1}$. The purpose of the following calculation is to do exactly that – trim the space needed for description of observed spectral vectors. Mathematically, this can be done by performing a *singular value decomposition* (SVD) on a sample correlation matrix given by

$$\widehat{\mathbf{R}}_y = [\mathbf{y}^{(1)}, \ldots, \mathbf{y}^{(N)}][\mathbf{y}^{(1)}, \ldots, \mathbf{y}^{(N)}]^T/N. \tag{2}$$

If the noise is assumed to have a zero-mean normal independent, as well as identical distributions for each $\mathbf{y}^{(\mathbf{i})} = \mathbf{x}^{(\mathbf{i})} + \mathbf{n}^{(\mathbf{i})}$, then the SVD leads to a following decomposition:

$$\widehat{\mathbf{R}}_y = \mathbf{E}\left(\mathbf{\Sigma} + \sigma_n^2 \mathbf{I}_L\right)\mathbf{E}^T \equiv \widehat{\mathbf{R}}_x + \widehat{\mathbf{R}}_n, \tag{3}$$

where $\mathbf{E} \equiv [\mathbf{e}_1, \ldots, \mathbf{e}_L]$ is a matrix of eigenvectors of $\widehat{\mathbf{R}}_y$, $\mathbf{\Sigma}$ is a matrix with eigenvalues of $\widehat{\mathbf{R}}_y$ on the diagonal, $\mathbf{I}_L$ is the identity matrix of size $L \times L$ and σ is the standard deviation of the added noise.

The end result of SVD is the identification of directions ($[\mathbf{e}_1, \ldots, \mathbf{e}_L]$ eigenvectors) with decreasing value of variance (the diagonal eigenvalues of $\mathbf{\Sigma}$ are automatically sorted from largest to smallest value).

1.2 Identification of the signal subspace

Given the permutation $\pi = \{i_1, \ldots, i_L\}$ of indices $i = 1, \ldots, L$, we can decompose the space $\mathbb{R}^L$ into two orthogonal subspaces: the k-dimensional subspace $\langle \mathbf{E}_k \rangle$ spanned by $\mathbf{E}_k \equiv [\mathbf{e}_{i_1}, \ldots, \mathbf{e}_{i_k}]$ and its orthogonal complement $\langle \mathbf{E}_k \rangle^\perp$ spanned by $\mathbf{E}_k^\perp \equiv [\mathbf{e}_{i_{k+1}}, \ldots, \mathbf{e}_{i_L}]$.

The observed spectral vector $\mathbf{y}$ (from now onwards, we are dropping indexing for clarity) can be projected into subspace $\langle \mathbf{E}_k \rangle$ by a projection matrix $\mathbf{U}_k = \mathbf{E}_k \mathbf{E}_k^T$:

$$\widehat{\mathbf{x}}_k \equiv \mathbf{U}_k \mathbf{y} \tag{4}$$

The conditional expected mean value of the projected vector $\widehat{\mathbf{x}}_k$ given condition $\mathbf{x}$ (signal $\mathbf{x}$ measured) is

$$\begin{aligned} \mathbb{E}[\widehat{\mathbf{x}}_k|\mathbf{x}] &= \mathbf{U}_k \mathbb{E}[\mathbf{y}|\mathbf{x}] \\ &= \mathbf{U}_k \mathbb{E}[\mathbf{x} + \mathbf{n}|\mathbf{x}] \\ &= \mathbf{U}_k \mathbf{x} \\ &\equiv \mathbf{x}_k \end{aligned} \tag{5}$$

where $\mathbb{E}[\cdot|\cdot]$ is a conditional expectation operator. In the above, we have taken advantage of

the fact that the expectation value of zero-mean normal noise distribution $\mathbb{E}[\mathbf{n}|\mathbf{x}] = 0$. The projection of the signal spectral vector $\mathbf{x}$ into subspace $\langle \mathbf{E}_k \rangle$ must be $\mathbf{x}_k$ by definition. This result is very intuitive. Upon projection of $\mathbf{y}$, we expect to get 'on average' $\mathbf{x}_k$, because the observed noise in the sample signal has zero mean.

The expected variance of the projection $\widehat{\mathbf{x}}_k$ given condition $\mathbf{x}$ is

$$\begin{aligned} \mathbb{E}[(\widehat{\mathbf{x}}_k - \mathbf{x}_k)(\widehat{\mathbf{x}}_k - \mathbf{x}_k)^T|\mathbf{x}] &= \mathbb{E}[(\mathbf{U}_k\mathbf{y} - \mathbf{U}_k\mathbf{x})(\mathbf{U}_k\mathbf{y} - \mathbf{U}_k\mathbf{x})^T|\mathbf{x}] \\ &= \mathbf{U}_k\mathbb{E}[\mathbf{n}\mathbf{n}^T|\mathbf{x}]\mathbf{U}_k^T \\ &= \mathbf{U}_k\widehat{\mathbf{R}}_n\mathbf{U}_k^T, \end{aligned} \tag{6}$$

where we have used $\mathbf{y} - \mathbf{x} = \mathbf{n}$.

We aim to identify signal subspace such that the 'difference' (error) between the original signal $\mathbf{x}$ and the projected signal $\widehat{\mathbf{x}}_k$ is negligible. To achieve this, we calculate the mean square error, mse, between the original $\mathbf{x}$ and its projection $\widehat{\mathbf{x}}_k$

$$\mathrm{mse}(k|\mathbf{x}) = \mathbb{E}[(\mathbf{x} - \widehat{\mathbf{x}}_k)^T(\mathbf{x} - \widehat{\mathbf{x}}_k)|\mathbf{x}]. \tag{7}$$

Using $\widehat{\mathbf{x}}_k = \mathbf{U}_k\mathbf{y} = \mathbf{U}_k(\mathbf{x} + \mathbf{n}) = \mathbf{x}_k + \mathbf{U}_k\mathbf{n}$, we can rewrite Eq. 7 as follows

$$\mathrm{mse}(k|\mathbf{x}) = \mathbb{E}[(\mathbf{x} - \mathbf{x}_k - \mathbf{U}_k\mathbf{n})^T(\mathbf{x} - \mathbf{x}_k - \mathbf{U}_k\mathbf{n})|\mathbf{x}]. \tag{8}$$

Clearly, $\mathbf{x} - \mathbf{x}_k$ is just an orthogonal complement of $\mathbf{x}_k$ – mathematically, $\mathbf{x} - \mathbf{x}_k = \mathbf{U}_k^{\perp}\mathbf{x}$. Furthermore, the projection operator satisfies $\mathbf{U}_k^2 = \mathbf{U}_k$ (applying projection to already projected vector has no further effect), and $\mathbf{U}_k = \mathbf{E}_k\mathbf{E}_k^T = \mathbf{E}_k^T\mathbf{E}_k = \mathbf{U}_k^T$ (valid for any orthogonal matrix (projection matrix is orthogonal)). Finally, the projection of the $\mathbf{x}$ into the original space is just an identity matrix $\mathbf{I}$, but any orthogonal projection can be written as a sum of projection into subspace and its orthogonal complement. Therefore, the following is also valid $\mathbf{I} = \mathbf{U}_k^{\perp} + \mathbf{U}_k$. We can therefore cast eq. (8) into the following form:

$$\begin{aligned} \mathrm{mse}(k) &= \mathbb{E}[(\mathbf{U}_k^{\perp}\mathbf{x})^T(\mathbf{U}_k^{\perp}\mathbf{x})] - \mathbb{E}[(\mathbf{U}_k^{\perp}\mathbf{x})^T]\overbrace{\mathbb{E}[(\mathbf{U}_k\mathbf{n})]}^{0} - \overbrace{\mathbb{E}[(\mathbf{U}_k\mathbf{n})^T]}^{0}\mathbb{E}[(\mathbf{U}_k^{\perp}\mathbf{x})] + \mathbb{E}[\mathbf{n}^T\mathbf{U}_k^T\mathbf{U}_k\mathbf{n}] \\ &= \mathbb{E}[\mathbf{x}^T(\mathbf{U}_k^{\perp})^T\mathbf{U}_k^{\perp}\mathbf{x}] + \mathbb{E}[\mathbf{n}^T\mathbf{U}_k^T\mathbf{U}_k\mathbf{n}]. \end{aligned} \tag{9}$$

We further use the following identity that holds for any two vectors $\mathbf{a} \equiv |a\rangle$ and $\mathbf{b} \equiv |b\rangle$ (Dirac's notation):

$$\mathbf{a}^T\mathbf{b} = \langle a|b\rangle = \sum_i \langle a|e_i\rangle\langle e_i|b\rangle = \sum_i \langle e_i|b\rangle\langle a|e_i\rangle \equiv \mathrm{tr}(\mathbf{b}\mathbf{a}^T) \tag{10}$$

and rewrite eq. (9) as

$$\mathrm{mse}(k) = \mathrm{tr}(\mathbf{U}_k^{\perp}\mathbf{x}\mathbf{x}^T(\mathbf{U}_k^{\perp})^T) + \mathrm{tr}(\mathbf{U}_k\mathbf{n}\mathbf{n}^T\mathbf{U}_k^T) \tag{11}$$

As the trace is invariant with respect to cyclic permutations of matrices $\mathrm{tr}(ABC) = \mathrm{tr}(CAB) =$

tr(BCA), we can further write:

$$\begin{aligned}\text{mse}(k) &= \text{tr}((\mathbf{U}_k^{\perp})^T\mathbf{U}_k^{\perp}\mathbf{x}\mathbf{x}^T) + \text{tr}(\mathbf{U}_k^T\mathbf{U}_k\mathbf{n}\mathbf{n}^T) \\ &= \text{tr}(\mathbf{U}_k^{\perp}\widehat{\mathbf{R}}_x) + \text{tr}(\mathbf{U}_k\widehat{\mathbf{R}}_n),\end{aligned} \tag{12}$$

where we have again used the identities $\mathbf{U}_k = \mathbf{U}_k^T$ and $\mathbf{U}_k^2 = \mathbf{U}_k$.

Finally, we use eq. (3) along with $\mathbf{I} = \mathbf{U}_k^{\perp} + \mathbf{U}_k$ to arrive at

$$\text{mse}(k) = \text{tr}(\mathbf{U}_k^{\perp}\widehat{\mathbf{R}}_y) + 2\,\text{tr}(\mathbf{U}_k\widehat{\mathbf{R}}_n) - \overbrace{\text{tr}(\widehat{\mathbf{R}}_n)}^{C}, \tag{13}$$

where C is a constant not relevant for further calculations.

The optimisation procedure for finding the signal subspace is based on the minimisation of eq. (13) with respect to all possible permutations $\pi = \{i_1, \ldots, i_L\}$ of subspaces with dimensions determined by k. The outcome of the optimisation procedure is therefore a subspace of dimensionality $\hat{k}$ along with the permutation $\hat{\pi}$ of the eigenvectors spanning this subspace:

$$(\hat{k}, \hat{\pi}) = \arg\min_{k,\pi}\{\mathbf{U}_k^{\perp}\widehat{\mathbf{R}}_y) + 2\,\text{tr}(\mathbf{U}_k\widehat{\mathbf{R}}_n\}, \tag{14}$$

where the dependence on the permutation π is through $\mathbf{U}_k = \mathbf{E}_k\mathbf{E}_k^T$. The first term in eq. (14) is related to the error of projection into subspace. This is a decreasing function of k. This is obvious because with each added dimension, the projection must become more accurate. In the extreme case of $k = L$ the projection is just identity with no projection error. The second term in eq. (14) is related to the noise power. This is an increasing function of k. The sum of decreasing and increasing function yields a unique global minimum. This minimum determines the point of smallest projection error along with the smallest signal noise.

1.3 Projection of sample onto 'signal subspace'

Let us denote the subspace determined by permutation $\hat{\pi}$ and dimension $\hat{k} = p$ as $\langle\mathbf{E}_p\rangle$. This subspace is spanned by vectors $\mathbf{E}_p \equiv [\mathbf{e}_1, \ldots \mathbf{e}_p]$ as determined in the previous section. Let $\mathbf{E}_p\mathbf{E}_p^T$ be a projection matrix. Then $(\mathbf{E}_p\mathbf{E}_p^T)\mathbf{y}$ is the orthogonal projection of $\mathbf{y} \in \mathbb{R}^L$ onto the subspace spanned by columns of $\mathbf{E}_p$. The $\mathbf{x} \equiv \mathbf{E}_p^T\mathbf{y}$ then denotes coordinates of vector $\mathbf{y}$ with respect to the orthonormal basis $[\mathbf{e}_1, \ldots \mathbf{e}_p]$.

2 Projection of subspace vectors into a hyperplane best fitting the data

In the ideal (no noise) situation, all spectral vectors lie in a *simplex* (generalisation of a triangle or tetrahedron to an arbitrary dimension) residing on a *hyperplane*[1] of dimension $\mathbb{R}^{p-1}$. This hyperplane (or affine set) is fully contained within the p-dimensional subspace identified above. This interesting fact stems from the assumption of linear mixing model:

$$\mathbf{y} = \mathbf{M}\mathbf{s}, \tag{15}$$

where $\mathbf{M} \equiv [\mathbf{m}_1, \dots, \mathbf{m}_p]$ is an $L \times p$ endmember matrix ($\mathbf{m}_j$ denotes the jth member signature), p is the number of endmembers present, and $\mathbf{s} = [s_1, \dots, s_p]^T$ is the abundance vector containing the fractions of each endmember. To be physically meaningful, the abundance fractions are subject to non-negativity and sum-to-one constraints:

$$s_j \geq 0, \qquad \sum_{j=1}^{p} s_j = 1. \tag{16}$$

From this it is clear that only $p-1$ components of $\mathbf{s}$ (and $\mathbf{y}$ for that matter) can be freely chosen, because the p-component is given by:

$$s_p = 1 - \sum_{j=1}^{p-1} s_j. \tag{17}$$

Or in other words, the spectral vectors have to lie on a hyperplane of dimension $p-1$ (the spectral vectors, in fact, lie within a $p-1$-dimensional simplex, but we only need the notion of a hyperplane for the next step.)

Due to spectral variability from pixel to pixel, modelling errors and noise, the observed spectral vectors $\mathbf{y}$, and therefore also their projection $\mathbf{x}$ (see Sec. 1.3), are not exactly on this hyperplane. As the following procedure requires all vectors to be in the hyperplane, we need to identify it and project vectors $\mathbf{x} \in \mathbb{R}^p$ onto it.

To project the vectors $\{\mathbf{x}^{(i)}, i = 1, \dots, N\}$ onto a hyperplane such that all the vectors projected into this hyperplane $\{\widetilde{\mathbf{x}}^{(i)}, i = 1, \dots, N\}$ represent the original[2] vectors $\{\mathbf{x}^{(i)}, i = 1, \dots, N\}$ as accurately as possible. This procedure is similar to fitting of a line through a set of points in two-dimensional space (linear regression) or to the fitting of a plane through a set of points in three-dimensional space. We can find the coefficients of such a plane by using the notion of the *least square method*. In principle, we minimize the variance of data in the direction perpendicular to the hyperplane. This leads to minimal error upon projection to hyperplane. Applying this to our problem, we find the representation of the projected vectors

[1]Hyperplane is a subspace that is one dimension smaller than the surrounding space (plane in the three-dimensional space is therefore also a hyperplane).

[2]Please note that these vectors are already projected into signal subspace.

in the hyperplane as [13];

$$\widetilde{\mathbf{x}}^{(i)} = \overline{\mathbf{x}} + \mathbf{U}_{p-1}\left(\mathbf{x}^{(i)} - \overline{\mathbf{x}}\right), \quad i = 1, \ldots, N \tag{18}$$

where $\overline{\mathbf{x}}$ is the sample average of vectors $\{\mathbf{x}^{(i)}, i = 1, \ldots, N\}$ ('center of mass' of the hyperplane). $\mathbf{U}_{p-1}$ is a projection matrix (projection into hyperplane minimizing the variance). $\mathbf{U}_{p-1}$ is a $p\times(p-1)$ orthonormal matrix with columns defined by $p-1$ eigenvectors of the sample covariance matrix of $\{\mathbf{x}^{(i)}, i = 1, \ldots, N\}$.

To summarise we have two projections with this scheme: The first projects the observed spectral vectors onto the identified subspace signal, and the second projects the already projected vectors onto the identified hyperplane (affine set). We stress that the effect of the two projections could not be obtained just by using the second one, at least in the case of non-white noise. This is because the optimal signal subspace estimate depends on the noise correlation matrix. Also note that even though we project into hyperplane, we still work in the p-dimensional space and not the $(p-1)$-dimensional space.

From now on we drop the tilde symbol from the vector projected to the hyperplane. We can now write the double-projected signal vectors as

$$\mathbf{x} = \mathbf{A}\mathbf{s}, \tag{19}$$

where $\mathbf{A} \in \mathbb{R}^{p\times p}$ is the endmember matrix and $\mathbf{s}$ is the abundance vector. The relation between the projected endmember matrix $\mathbf{A}$ and the original endmember matrix $\mathbf{M}$ is then given by:

$$\mathbf{M} = \mathbf{E}_p\mathbf{A}. \tag{20}$$

2.1 The case of zero noise

It is interesting to examine the case when the noise matrix is strictly zero. In this case the vectors $\mathbf{x^{(i)}}$ are a linear combination of endmembers vectors (spectra) $\mathbf{m_j}$.

$$\mathbf{x^{(i)}} = \sum_{j=1}^{p} s_{ij}\mathbf{m_j} \tag{21}$$

We will now apply the HySime algorithm detailed previously to this particular dataset [13]. Of course in this case the noise correlation matrix $\mathbf{R_n}$ is zero, so we will be minimising only the first part in Equation 14 that is

$$(\hat{k}, \hat{\pi}) = \arg\min_{k,\pi}\{\mathbf{U}_k^{\perp}\widehat{\mathbf{R}}_x) \tag{22}$$

Here we substituted the signal correlation matrix $\mathbf{R}_y$ by $\mathbf{R}_x$ (because now $\mathbf{y} = \mathbf{x}$). In this case the signal correlation matrix $\mathbf{R}_x$ can be explicitly calculated to be:

$$\mathbf{R}_x = \frac{1}{N}\sum_i \left(\mathbf{x^{(i)}} - \overline{\mathbf{x}}\right)\left(\mathbf{x^{(i)}} - \overline{\mathbf{x}}\right)^T \tag{23}$$

Here,

$$\overline{\mathbf{x}} = \sum_{j=1}^{p} \overline{s_j}\mathbf{m_j} \tag{24}$$

where

$$\overline{s_j} = \frac{1}{N}\sum_i s_{ji} \tag{25}$$

With that we have:

$$\mathbf{x^{(i)}} - \overline{\mathbf{x}} = \sum_{j=1}^{p} \left(s_{ij} - \overline{s_j}\right)\mathbf{m_j} \tag{26}$$

Consequently,

$$\mathbf{R}_x = \frac{1}{N}\sum_i \sum_{j=1}^{p} \left(s_{ij} - \overline{s_j}\right)^2 \mathbf{m_j}\mathbf{m_j}^T = \frac{1}{N}\sum_{j=1}^{p}\left[\sum_i \left(s_{ij} - \overline{s_j}\right)^2\right]\mathbf{m_j}\mathbf{m_j}^T = \sum_{j=1}^{p}\alpha_j \mathbf{m_j}\mathbf{m_j}^T \tag{27}$$

Thus the correlation matrix for $\mathbf{x}$ is the sum of projection operators onto endmember vectors. Consequently if we apply the correlation matrix (operator) to an arbitrary vector $\mathbf{v}$, then the result lies in a p-dimensional space span by the vectors $\mathbf{m_j}$, because p linearly independent vectors span a p-dimensional space.

Now we need to apply to this correlation matrix a projection onto the affine subspace, $\mathbf{U}_k^{\perp} = \mathbf{E_k}\mathbf{E_k^T}$ span by a selection of its own eigenvectors $\mathbf{e_j}$, where k denotes a permutation. We then need to calculate the trace and then to find its minimum, in the following sequence. First for each fixed permutation we minimise with respect to k, for each permutation separately, and then we minimise with respect to permutations. To this aim we note that

$$\mathbf{U}_k^{\perp}\mathbf{R_x}\mathbf{e_j} = \begin{cases} \lambda_j e_j & \text{if} \quad j \geq k, \\ \\ 0 & \text{if} \quad j < k. \end{cases} \tag{28}$$

We can easily see that the trace of this operator is the sum of the eigenvalues of the covariance operator. The eigenvalues of the covariance operator are variances and they are all positive, This means that the trace of $\mathbf{U}_k^{\perp}\mathbf{R_x}$ is a decreasing function of k. Hence the minimum in each permutation is achieved for the largest possible k. However we note that all vectors $\mathbf{R_x}\mathbf{e_j}$ must lie in a p-dimensional space (the range of the covariance operator), as shown above. This means that the largest possible k is p. Now we minimise over all permutations, but the same answer, p is obtained for each permutation.

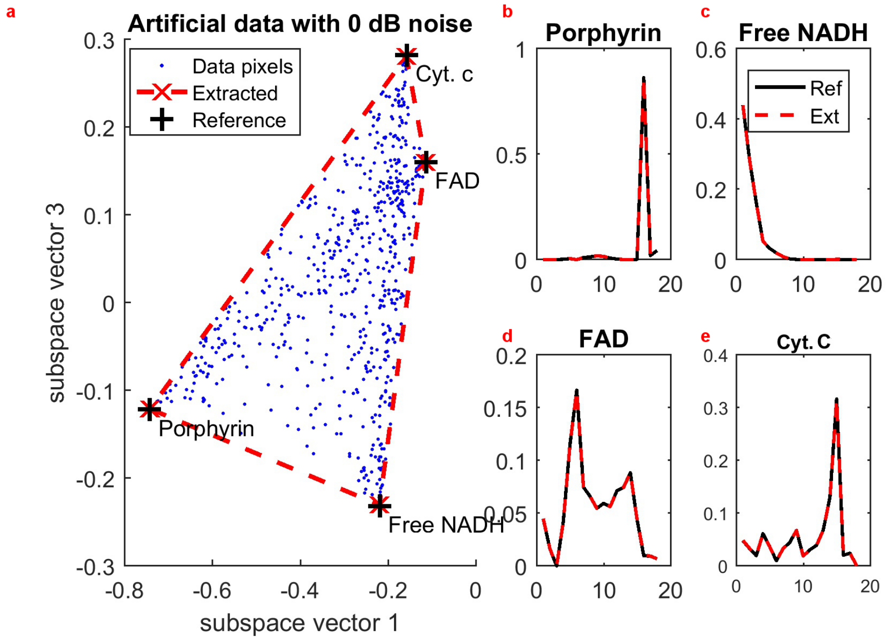

Figure 1: Simplex representation of artificial data without any noise

We therefore conclude that in the case of no noise the HySime returns the number of the endmembers [13]. It can be anticipated that in the case of small noise amplitude we will be getting exactly the same answer due to the noise covariance making only a small contribution to the minimisation. Figure (1) shows the simplex representation of artificial data without any noise. Here data pixels are presented with blue coloured dots and extracted fluorophores are with red coloured cross. Free NADH, Porphyrin, FAD and Cyt. c are notified with their corresponding marker. The reference spectra for Free NADH, Porphyrin, FAD and Cyt. C are shown as black coloured cross. And both reference spectra and extracted spectra are coincident due to zero noise.

3 DECA algorithm

In this work, Robust Dependent Component Analysis (RoDECA) algorithm is used for extracting the native fluorophores and their corresponding abundance vector, a modified version of Dependent Component Analysis (DECA) algorithm [14]. DECA is an unsupervised hyperspectral unmixing method based on the assumption of the linear mixing model, which is well suited for highly mixed datasets, where there are no pure pixel presents. In this section we will discuss about the DECA approaches.

3.1 DECA approach

The DECA analysis begins with the initial blind estimation of the endmember matrix by modelling the fractional abundance vector $\mathbf{s}$ as a mixture of Dirichlet densities [14].

The authors in [14] assumed that $\mathbf{W} \equiv \mathbf{A}^{(-1)}$. Then, they [14] had $\mathbf{s} = \mathbf{Wx}$ [14]. The main advantage of using the Dirichlet densities is that this approach automatically enforces the non-negativity and sum-to-one constraints, and thus, it accounts for statistical dependence usually found in hyperspectral data which satisfy the LMM conditions. Moreover, the Dirichlet densities are well suited to model the complex distributions in which the 'mass probability function' is scattered over several clusters inside the simplex [11, 14]. In the following, we extensively draw on [14] including using their notations.

"

$$
\begin{aligned}
p_S(\mathbf{s}|\theta) &\equiv \sum_{q=1}^{k} \epsilon_q D(\mathbf{s} \mid \theta_{\mathbf{q}}), \\
&= \sum_{q=1}^{k} \epsilon_q \frac{\Gamma\left(\sum_{j=1}^{p} \theta_q j\right)}{\prod_{j=1}^{p} \Gamma(\theta_q j)} \prod_{j=1}^{p} s_j^{\theta_q j - 1},
\end{aligned}
\tag{29}
$$

where for $q = 1 \ldots, k$, ϵ_q and $D(\mathbf{s} \mid \theta_{\mathbf{q}})$ denotes the probability of mode q and its Dirichlet density with the parameter $\boldsymbol{\theta}_q \equiv \{\theta_{q1}, \ldots, \theta_{qp}\}$ [14]. They [14] denoted the complete set of the parameters as $\boldsymbol{\theta} \equiv \{\epsilon_1, \ldots, \epsilon_k, \boldsymbol{\theta}_1, \ldots, \boldsymbol{\theta}_k\}$ [14]. Since the spectral vectors x plays now the role of the observed data, they must based their inferences on their density, denoted by p_X [14], which in terms of p_S and given the linear and invertible relationship $\mathbf{s} = \mathbf{Wx}$ between s and x, is given by,

$$
p_X(\mathbf{x}|\mathbf{W}, \boldsymbol{\theta}) = p_S(\mathbf{s} = \mathbf{Wx}|\boldsymbol{\theta}) \,|\det(\mathbf{W}). \tag{30}
$$

3.2 Maximum Likelihood Estimation

Consider that each vector x represents one particular outcome of a $p-$dimensional random variable $\mathbf{X} = [X_1, \ldots, X_p]^{\mathrm{T}}$ [14]. Given a set of N independent and identically distributed (i.i.d.) samples $\mathcal{X} \equiv [\mathrm{x}^{(1)}, \ldots, \mathrm{x}^{(N)}]$, the log-likelihood of the set of

the parameters $\boldsymbol{\theta}$ and endmember matrix $\mathbf{W}$ is

$$\begin{aligned}
\mathcal{L}(\mathbf{W},\boldsymbol{\theta}) &\equiv \log p_X(\mathcal{X}|\mathbf{W},\boldsymbol{\theta}) && (31)\\
&= \sum_{i=1}^{N}\left[\log p_X\left(\mathbf{x}^{(i)}|\mathbf{W},\boldsymbol{\theta}\right)\right] && (32)\\
&= \sum_{i=1}^{N}\left[\log p_S\left(\mathbf{s}^{(i)}|\boldsymbol{\theta}\right)\right] + N\log|\det\mathbf{W}| && (33)\\
&= \sum_{i=1}^{N}\left[\log\sum_{q=1}^{k}\epsilon_q D\left(\mathbf{s}^{(i)} \mid \theta_{\mathbf{q}}\right)\right] + N\log|\det\mathbf{W}| && (34)
\end{aligned}$$

where,

$$\mathbf{s}^{(i)} \equiv \mathbf{W}\mathbf{x}^{(i)} \qquad (35)$$

Then the maximum likelihood estimate

$$\left(\widehat{\mathbf{W}},\widehat{\boldsymbol{\theta}}\right)_{\mathrm{ML}} \equiv \arg\max_{\mathbf{W},\boldsymbol{\theta}} \mathcal{L}(\mathbf{W},\boldsymbol{\theta}) \qquad (36)$$

can not be found analytically [5, 10]. However, the *expectation maximization (EM)* framework can be used to obtain the maximum likelihood estimate [9]. This framework relies on the incomplete data and the missing data. In this set-up, $\mathcal{X}$ is the incomplete data and $\mathcal{Z}$ is the missing data, where missing data denoted by;

$$\mathcal{Z} \equiv \{\mathbf{z}^{(i)},\ldots,\mathbf{z}^{(N)}\}, \qquad (37)$$

are the set of N $k-$ dimensional vectors representing which component has produced each sample. Each vector $\mathbf{z}^{(i)} = [z_1^{(i)},\ldots,z_k^{(i)}]$ is a binary $k-$vector, where only one component $z_q^{(i)}$is set to one indicating which mode produced the $i-$sample. Then the complete log-likelihood is given by,

$$\begin{aligned}
\mathcal{L}_C(\boldsymbol{\theta},\mathbf{W}) &\equiv \log\ [\ p_{X,Z}(\mathcal{X},\mathcal{Z}|\boldsymbol{\theta})] + N\log|\det\mathbf{W}|\\
&= \sum_{i=1}^{N}\left[\ \sum_{q=1}^{k} z_q^{(i)}\log\ \left(\epsilon_q D\left(\mathbf{s}^{(i)} \mid \theta_{\mathbf{q}}\right)\right)\right] + N\log|\det\mathbf{W}| && (38)
\end{aligned}$$

The EM algorithm iterates between the *Estimation* steps and the *Maximisation* step [8, 9].

3.3 Estimation Step

It computes the conditional expectation of the complete log-likelihood, given the samples and the current estimate $\widehat{\boldsymbol{\theta}}^{(t)}$ [14]. The result is so-called Q-function

$$Q\left(\boldsymbol{\theta},\mathbf{W};\ \widehat{\boldsymbol{\theta}}^{(t)},\widehat{\mathbf{W}}^{(t)}\right)=N\ \log\ |\det\mathbf{W}|+\sum_{i=1}^{N}\left[\ \sum_{q=1}^{k}\beta_q^{(i,t)}\ \log\ \left(\epsilon_q D\left(\mathbf{s}^{(i,t)}\mid\theta_{\mathbf{q}}\right)\right)\right] \tag{39}$$

where,

$$\beta_q^{(i,t)}\ \equiv\ \mathbb{E}\ \left[z_q^{(i)}|\ \widehat{\boldsymbol{\theta}}^{(t)}\right] \tag{40}$$

$$=\ \frac{\widehat{\epsilon_q}^{(t)}D\left(\mathbf{s}^{(i,t)}|\widehat{\boldsymbol{\theta}}_q^{(t)}\right)}{\sum_{l=1}^{k}\widehat{\epsilon_l}^{(t)}D\left(\mathbf{s}^{(i,t)}|\widehat{\boldsymbol{\theta}}_l^{(t)}\right)} \tag{41}$$

with $\mathbf{s}^{(i,t)}\equiv\widehat{\mathbf{W}}^{(t)}\ \widehat{\mathbf{x}}^{(i)}$ and $\mathbb{E}(\cdot)$ denote the mean value operator [14].

3.4 Maximization Step

It updates the parameter estimates are updated according to

$$\left(\widehat{\boldsymbol{\theta}}^{(t+1)},\widehat{\mathbf{W}}^{(t+1)}\right)=\arg\max_{\boldsymbol{\theta},\mathbf{W}}\left\{Q\left(\boldsymbol{\theta},\mathbf{W};\ \widehat{\boldsymbol{\theta}}^{(t)},\widehat{\mathbf{W}}^{(t)}\right)\right\} \tag{42}$$

Optimization of Eq. (42) is still a hard problem. Instead of solving it exactly, they [14] implement alternative minimization with respect to $\boldsymbol{\theta}$ and to $\mathbf{W}$. In the same vein, instead of computing $\widehat{\boldsymbol{\theta}}^{(t+1)}$, they [14] maximize the $Q-$ function with respect to $\widehat{\theta}_{qj}$, for $j=1,\ldots,p$, resulting in the following learning rules for mixing probabilities and for the mixture of Dirichlet source parameters [12].

$$\epsilon_q^{(t+1)}=\frac{1}{N}\sum_{i=1}^{N}\beta_q^{(i,t)}, \tag{43}$$

$$\widehat{\theta}_{qj}^{(t+1)}\ =\Psi^{(-1)}\left(\Psi\left(\sum_{l=1}^{p}\widehat{\theta}_{ql}^{t}\right)+\frac{\sum_{i=1}^{N}\left[\beta_q^{(i,t)}+\log\ \widehat{s_j}^{(i,t)}\right]}{\sum_{i=1}^{N}\left(\beta_q^{(i,t)}\right)}\right) \tag{44}$$

for $q=1,\ldots,k$ and $k=1,\ldots,p$, where $\Psi(x)\equiv\frac{d}{dx}$ $(\log\Gamma(x))$ is the psi $(\cdot)$ function and the $\Psi^{-1}(\cdot)$ denotes its inverse.

3.5 Optimizing the nonconvex term; log $|\det(\mathbf{W})|$

Denoting $\mathcal{S}\equiv\mathbf{W}\mathcal{X}=[\mathbf{s}^{(1)},\ldots,\mathbf{s}^{(N)}]$, the optimization with respect to $\mathbf{W}$ amounts to compute

$$\widehat{\mathbf{W}}^{(t+1)}=\arg\max_{\mathbf{W}}\ \phi\ (\mathbf{W}\mathcal{X})+\log\ |\det(\mathbf{W})|, \tag{45}$$

s.t.:

$$\mathbf{W}\mathcal{X} \succeq 0 \qquad \mathbf{1}^T\mathbf{W}\mathcal{X} = \mathbf{1}_N^T \tag{46}$$

Here, $\phi(\mathbf{W}\mathcal{X})$ is defined in Equation 15 of Reference [14], and the constraints $\mathbf{W}\mathcal{X} \succeq 0$ and $\mathbf{1}^T\mathbf{W}\mathcal{X} = \mathbf{1}_N^T$ enforce, respectively, non-negativity and sum-to-one on each abundance fraction vector ($\mathbf{1}_p$ stands for $p-$dimensional column vector of ones). which is equivalent to:

$$\widehat{\mathbf{W}}^{(t+1)} = \arg\min_{\mathbf{W}} \ \phi\left(\mathbf{W}\mathcal{X}\right) - \log\left|\det(\mathbf{W})\right|, \tag{47}$$

where,

$$\mathbf{W}\mathcal{X} \succeq 0 \qquad \mathbf{1}_p^T\mathbf{W} = \mathbf{a}^T \tag{48}$$

$$\mathbf{a}^T = \mathbf{1}_N\mathcal{X}^T(\mathcal{X}\mathcal{X}^T)^{-1} \tag{49}$$

"

4 Modifications for RoDECA in this work

The problem of optimization in Eq. (47) has been solved in ref [14] by using *Dirichlet mixture unmixing via split augmented Lagrangian* (DUSAL). In the case of biological data with comparatively high levels of noise (lower SNR) the intermediate matrices $\mathbf{W}$ produced during the iterative procedure have negative elements. The DUSAL procedure, through its use of the $-\phi(\mathbf{W}\mathcal{X})$ function forces these negative elements to become zero at subsequent stages of iteration. By doing so, DUSAL eventually produces the matrix $\mathbf{W}$ which can no longer be inverted (from some point onwards in the iteration). However $\mathbf{W}$ must be able to be inverted because, as we indicated earlier, the matrix $\mathbf{A} = \mathbf{W}^{-1}$.

To overcome this problem, instead of DUSAL we introduced a more robust *Splitting and augmented Lagrangian* (SISAL) method.

4.1 Introduction of a soft negative function by SISAL

We used the previously developed method of simplex identification via the split augmented Lagrangian (SISAL) [3] to carry out the minimization of the argument in eq. (47), with respect to $\mathbf{W}$. In SISAL, the hard constraints of DUSAL (zeros in $\mathbf{W}$) are replaced by hinge type soft constraints [3], whose strength is controlled by a regularization parameter [3,4].

The SISAL algorithm arrives at near optimal solutions for the by using the hinge function [2,6]. The hinge function is applied just to the negative elements of $\mathbf{W}\mathcal{X}$ replacing them with small positive numbers and this is avoiding the creation of intermediate matrices $\mathbf{W}$ with excessive number of zeros like in DUSAL.

The spectra extracted with the help of SISAL are shown in Figure 2a. The extracted spectra however small negative values in some channels, because there is nothing in the SISAL method (as well as in DUSAL) to prevent such negative values. However, such negative fluorescence intensities are not physically possible. To address this issue, it is necessary to introduce an additional step to yield spectra with only positive values.

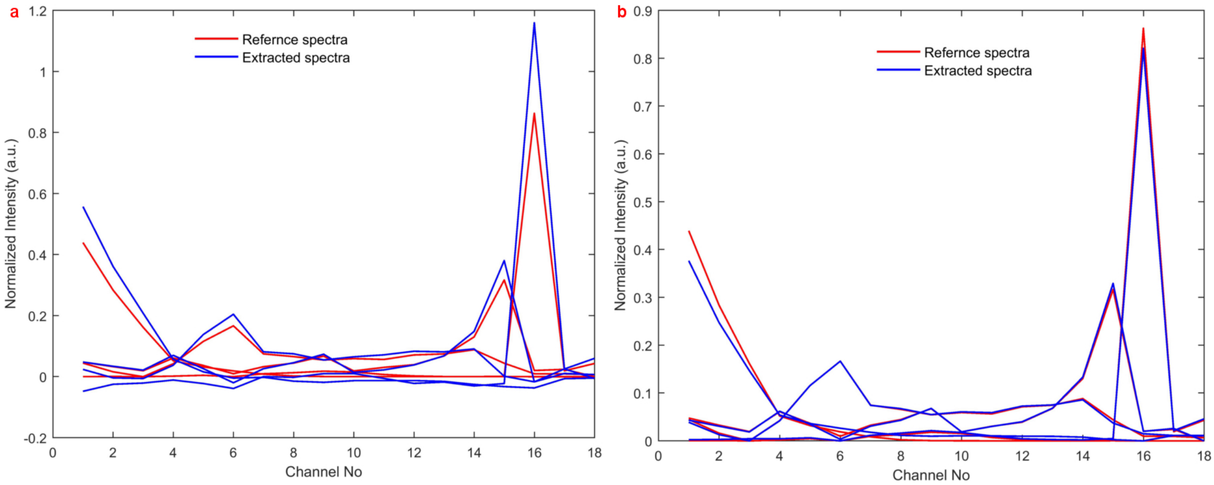

Figure 2: (a) Extracted endmember's spectra by using SISAL. Here four extracted spectra are presented in blue colour and the reference spectra are shown in solid red color with respect to the channel number. (b) Normalized value of extracted spectra. Here extracted spectra are shown in blue colour and the reference spectra are shown in solid red color after normalization.

4.2 Normalization of the spectra

In this work, we must ensure all fluorescence intensities in the extracted spectra are positive. Secondly, the extracted spectra must be normalised because input spectra have been normalised [1, 7].

The positivity of the spectra is enforced by simply shifting up each extracted spectrum by its minimum value. After that, this condition needs to be carried through the entire iterative EM algorithm.

Thus, we must project $\mathbf{W}$ onto $\mathbf{M}\left(=\mathbf{E}_p\mathbf{W}^{(-1)}\right)$ to ensure the positive and normalized fluorescence intensity. At each step of iteration the corresponding $\mathbf{M}$ is modified as follows;

$$\mathbf{M_{int}}' = \mathbf{M_{int}} - min\left(\mathbf{M_{int}}\right) \tag{50}$$

where, $\mathbf{M_{int}}$ is an intermediate matrix of endmember spectra, $\mathbf{M_{int}}'$ is the rescaled matrix of endmember spectra. It is needed to remember that $\mathbf{M_{int}}$ has $L \times p$ dimension, where $\mathbf{W}$ is the $p \times p$ matrix.

After that, we enforce the unitary value of each spectrum by dividing each element by

the Euclidean length of the vector by,

$$\mathbf{M_{int}}'' = \frac{\mathbf{M_{int}}'}{\sum_{k=1}^{L} \mathbf{M_{int}}'} \tag{51}$$

where, $\mathbf{M_{int}}''$ is the normalized-positive matrix of endmember spectra and $\mathbf{M_{int}}'$ is the positive matrix of endmember spectra through Eq. 51. Subsequently $\mathbf{M_{int}}''$ is again converted into $\mathbf{W}$ and this $\mathbf{W}$ is used for further calculation in the EM algorithm. We calculate the final $L \times p$ matrix $\mathbf{M}$ by;

$$\mathbf{M} = \mathbf{E}_p \mathbf{W}^{(-1)} \tag{52}$$

5 List of spectral channels

The presented list of the channels are used for the artificial data analysis. Whether, the retina cell analysis was conducted with only 16 channels, excluding the channel no 3 and channel no 6. [3]

Table 1: The list of spectral channels and the respective specification

Spectral channel number	Excitation wavelength ± 5 (nm)	Emission wavelength (bandwidth) (nm)	Dichroic mirror long pass (nm)	Power at objective (μW)	Quantum efficiency (QE) (unit less)	Subpanel serial in Figure 1 (f-w)
1	334	447 (60)	409	0.046	0.5	f
2	365	447 (60)	409	6.5	0.5	g
3*	375*	447 (60)	409	2.83	0.5	h
4	334	587 (35)	532	0.02	0.65	i
5	365	587 (35)	532	6.4	0.65	j
6*	375*	587 (35)	532	10.51	0.65	k
7	385	587 (35)	532	17.77	0.65	l
8	395	587 (35)	532	13.33	0.65	m
9	405	587 (35)	532	9.39	0.65	n
10	415	587 (35)	532	22.4	0.65	o
11	425	587 (35)	532	20.9	0.65	p
12	435	587 (35)	532	27.5	0.65	q
13	455	587 (35)	532	14.8	0.65	r
14	475	587 (35)	532	42.8	0.65	s
15	495	587 (35)	532	0.01	0.65	t
16	405	700 (long pass)	635	9.5	0.63	u
17	455	700 (long pass)	635	15.09	0.63	v
18	495	700 (long pass)	635	9.97	0.63	w

[3] *The excitation source of 375 nm was not stable during the retina cell data acquisition.

Table 2: Range of the colorbar values presented in figure 1 (f-w)

Spectral channel number	Subpanel serial in Figure 1 (f-w)	maximum value	minimum value
1	f	0.8065	0.0003
2	g	0.2837	0.0007
3	h	0.1618	0.0000
4	i	0.0611	0.0018
5	j	0.0368	0.0027
6	k	0.0189	0.0000
7	l	0.0327	0.0011
8	m	0.0446	0.0010
9	n	0.0674	0.0005
10	o	0.0188	0.0002
11	p	0.0305	0.0001
12	q	0.0392	0.0001
13	r	0.0679	0.0000
14	s	0.1303	0.0000
15	t	0.3167	0.0000
16	u	0.8628	0.0000
18	y	0.0241	0.0000
18	w	0.0432	0.0000

6 Supplementary Images

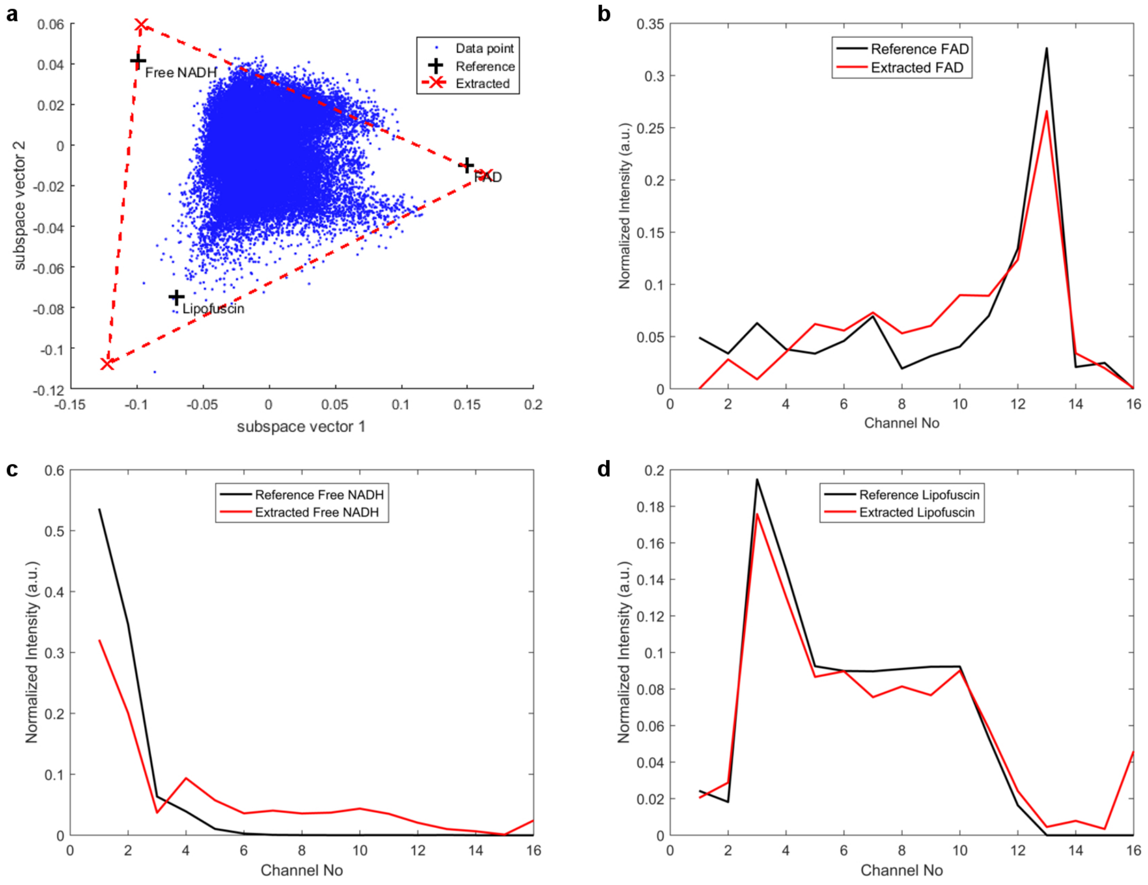

Figure 3: (a) Simplex representation of the retina data. Data pixels are presented with blue coloured dots and extracted fluorophores are with red coloured cross. Free NADH, FAD and lipofuscin are notified with their corresponding marker with the red dotted enclosure. The reference spectra for free NADH, FAD and lipofuscin are shown as black coloured cross. Extracted and reference spectra marked with red colour and black colour respectively for (b) FAD, (c) Free NADH and (d) Lipofuscin. Here the spectra are presented with respect to spectral channels, whereas in Figure 4 (a), (e) and (i) in main article are shown as a function of excitation. The spectra are shown with the excitation wavelength is using limited number of the channels.

6.1 Artificial Data-Case A: presenting in main article

We generated a pre-determined abundance map s (Figure 4 (a-d)), by creating 4 two-dimensional images N=24 $\times$ 25 pixel representing maps of abundance fractions and assigned them to each of the four chosen fluorophores NADH, FAD, porphyrin and cytochrome complex, Cyt. C (Figure 4 e).

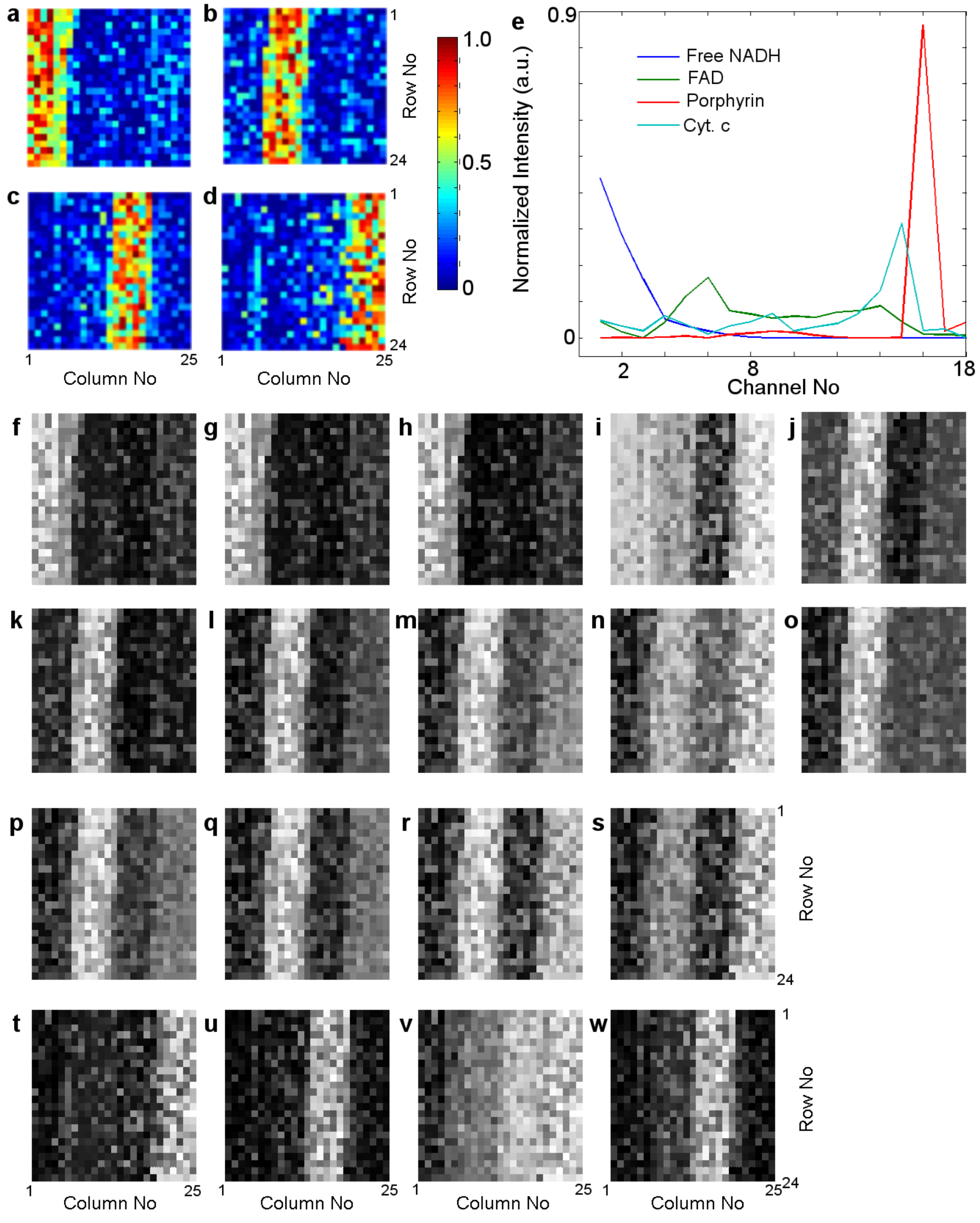

Figure 4: The artificial abundance map $\mathbf{s}_{[\mathbf{4}\times\mathbf{600}]}$ (for case A) of the total of 600 (24×25) pixels containing the fractions of each of four fluorophore (a) free NADH, (b) FAD, (c) porphyrin and (d) Cyt. C is presented. (e) The normalized reference spectra are shown for free NADH (blue), FAD (green), Cyt. C (cyan) and porphyrin (red). (f-w) Simulated hyperspectral image set (y=Ms+n) assuming zero noise (n=0) taken in L=18 different spectral channels. Images (f-w) are rescaled for better visualization as a grey colour-map, where the minimum value is displayed as black, and the maximum value (varying at each channel) is displayed as white.

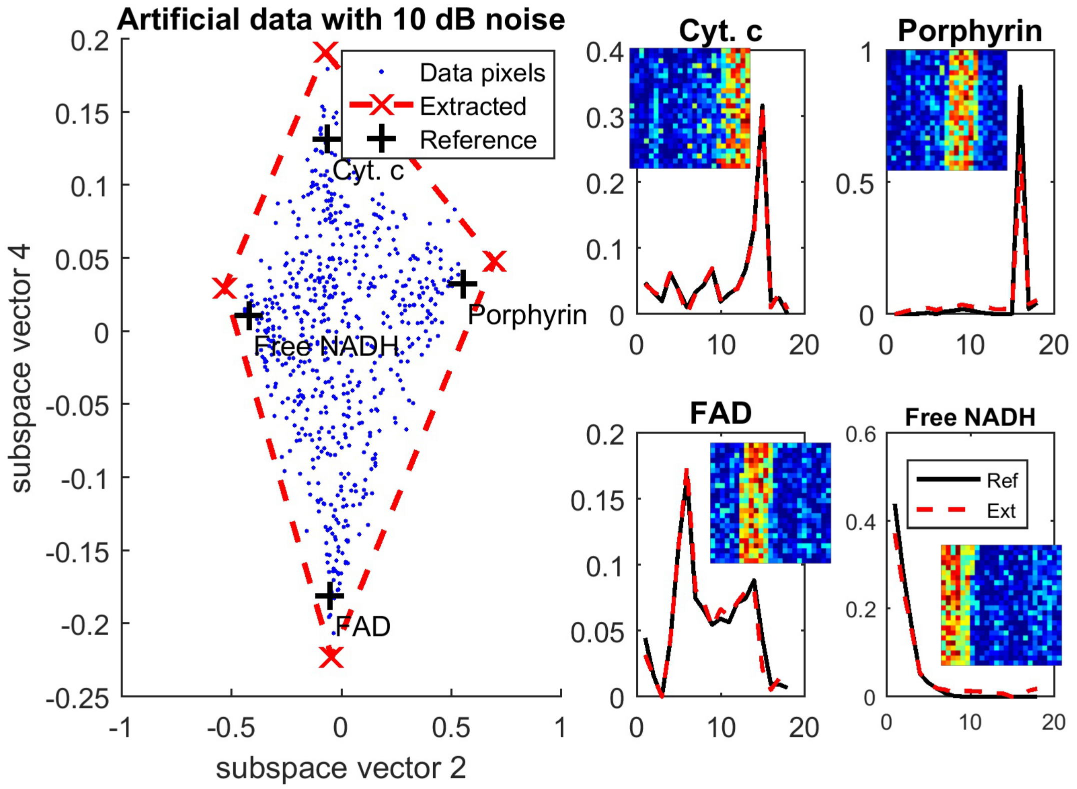

Figure 5: (clockwise)(first) Simplex representation of artificial data with SNR of 10 dB. Blue dots represent 600 data points. Red crosses represent the extracted endmembers by unsupervised unmixing with red dotted enclosure. Reference spectra of free NADH, FAD, Cyt. C and Porphyrin are shown by black crosses. (second-fifth) Extracted and reference spectra marked with red colour and black colour respectively for free NADH, FAD, Cyt. C and Porphyrin. Here the spectra are presented with respect to spectral channels.

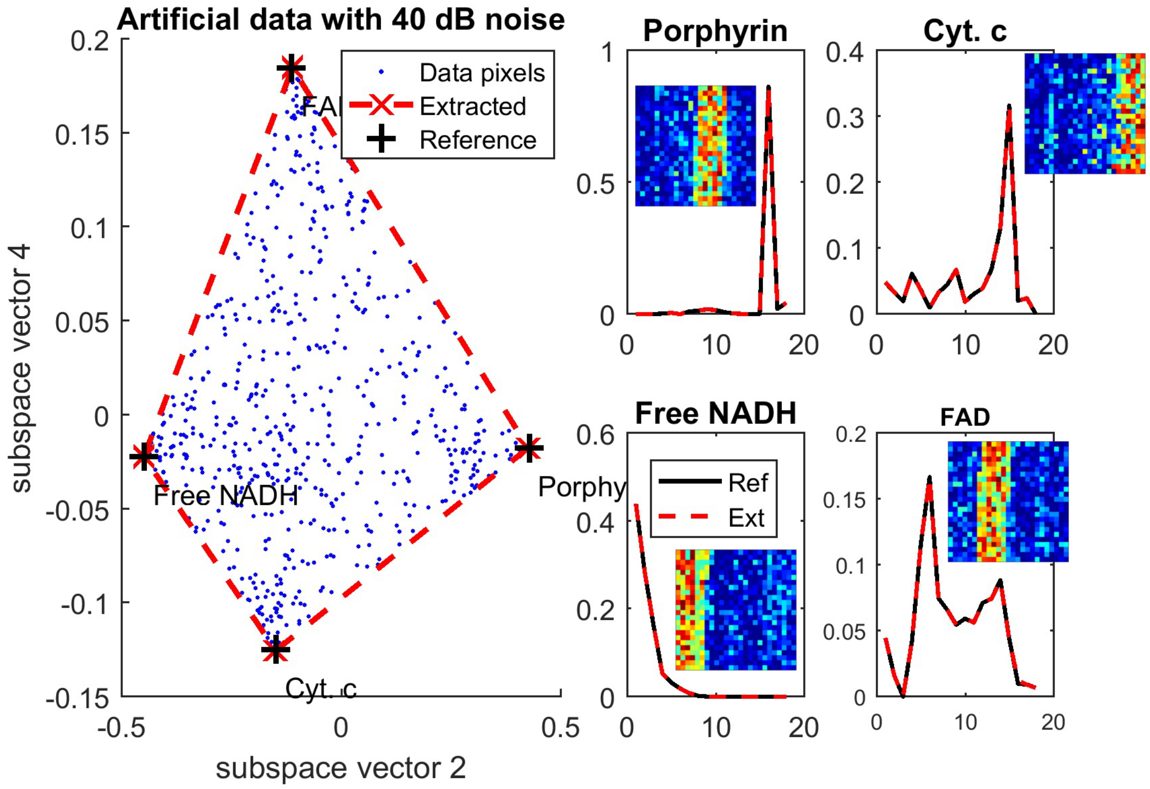

Figure 6: (clockwise)(first) Simplex representation of artificial data with SNR of 40 dB. Blue dots represent 600 data points. Red crosses represent the extracted endmembers by unsupervised unmixing with red dotted enclosure. Reference spectra of free NADH, FAD, Cyt. C and Porphyrin are shown by black crosses. (second-fifth) Extracted and reference spectra marked with red colour and black colour respectively for free NADH, FAD, Cyt. C and Porphyrin. Here the spectra are presented with respect to spectral channels.

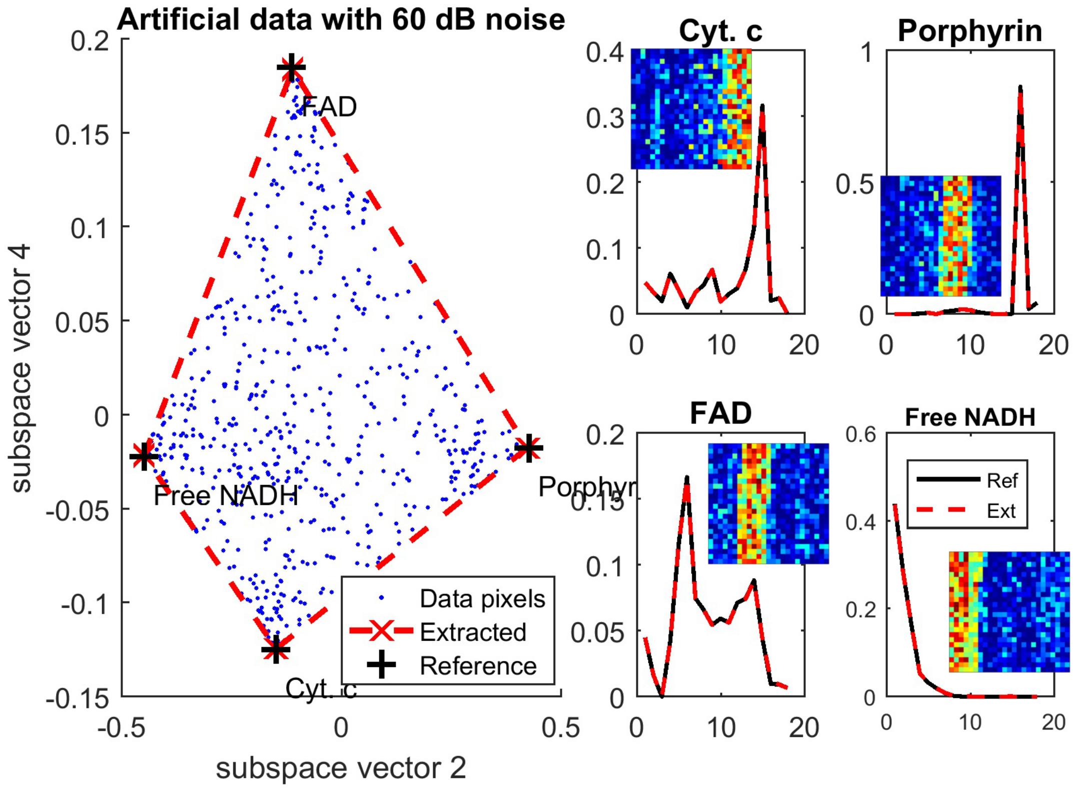

Figure 7: (clockwise)(first) Simplex representation of artificial data with SNR of 60 dB. Blue dots represent 600 data points. Red crosses represent the extracted endmembers by unsupervised unmixing with red dotted enclosure. Reference spectra of free NADH, FAD, Cyt. C and Porphyrin are shown by black crosses. (second-fifth) Extracted and reference spectra marked with red colour and black colour respectively for free NADH, FAD, Cyt. C and Porphyrin. Here the spectra are presented with respect to spectral channels.

6.2 Artificial Data- Case B

We generated a pre-determined abundance map s (Figure 8 (a-d)), by creating 4 two-dimensional images N=24 $\times$ 25 pixel representing maps of abundance fractions and assigned them to each of the four chosen fluorophores NADH, FAD, porphyrin and cytochrome complex, Cyt. C (Figure 8 e). In this case,FAD and porphyrin are overlapped at column 11 to column 16 with abundance fraction of around 50% between FAD and porphyrin.

In order to establish the robustness of RoDECA unmixing, this unmixing analysis was carried over with a broad range of added Gaussian white noise with SNR from 10 dB to 60 dB. The simplexes of the artificial data, as well as the spectra of extracted fluorophores compared with reference spectra for the SNR of 10 dB, 20 dB, 40 dB and 60 dB, are shown in Figures 9, 10, 11 and 12 respectively.

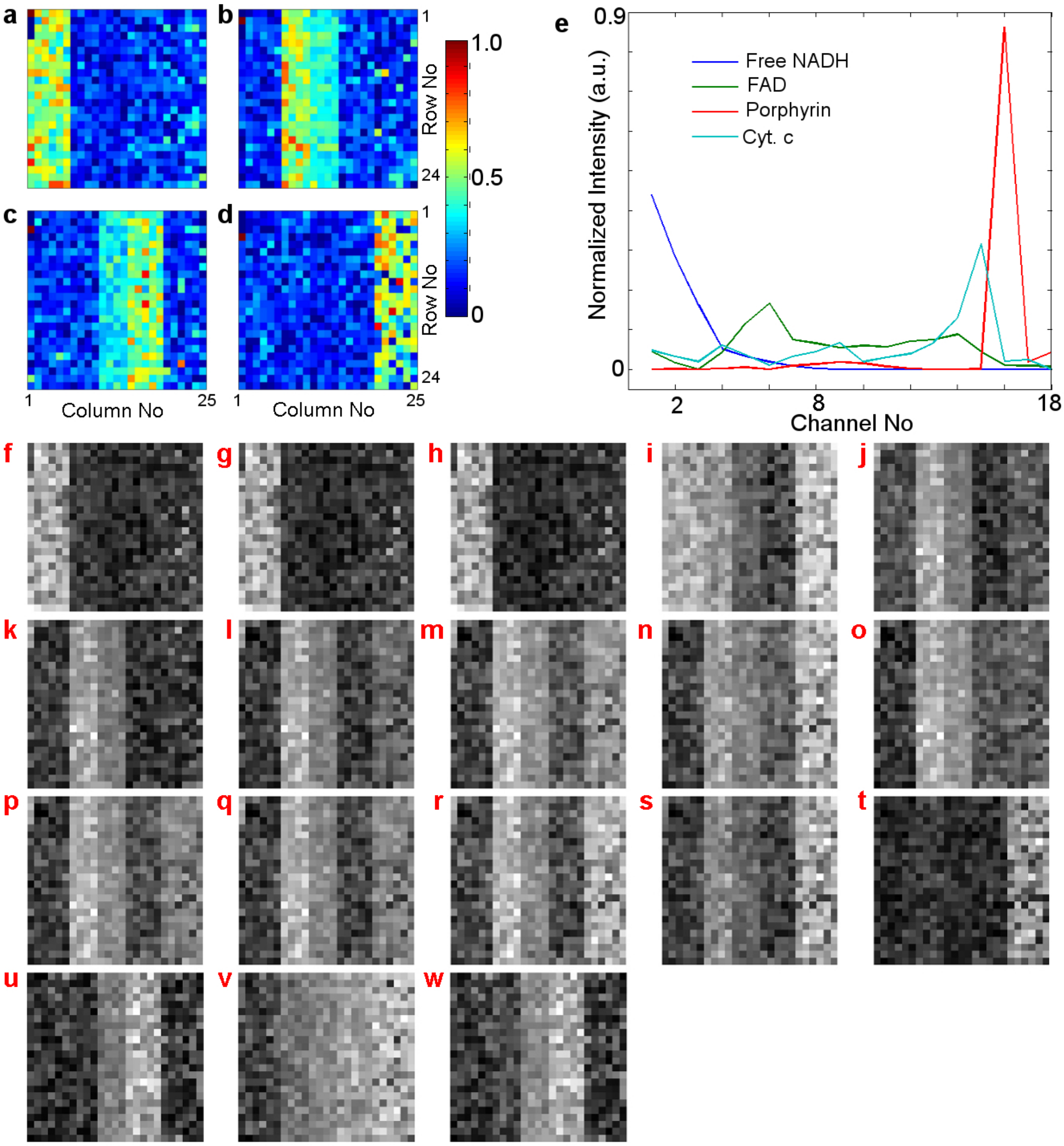

Figure 8: The artificial abundance map $\mathbf{s}_{[4\times 600]}$ (for case B) of the total of 600 (24 × 25) pixels containing the fractions of each of four fluorophore (a) free NADH, (b) FAD, (c) porphyrin and (d) Cyt. C is presented. (e) The normalized reference spectra are shown for free NADH (blue), FAD (green), Cyt. C (cyan) and porphyrin (red). (f-w) Simulated hyperspectral image set (y=Ms+n) assuming zero noise (n=0) taken in L=18 different spectral channels. Images (f-w) are rescaled for better visualization as a grey colour-map, where the minimum value is displayed as black, and the maximum value (varying at each channel) is displayed as white.

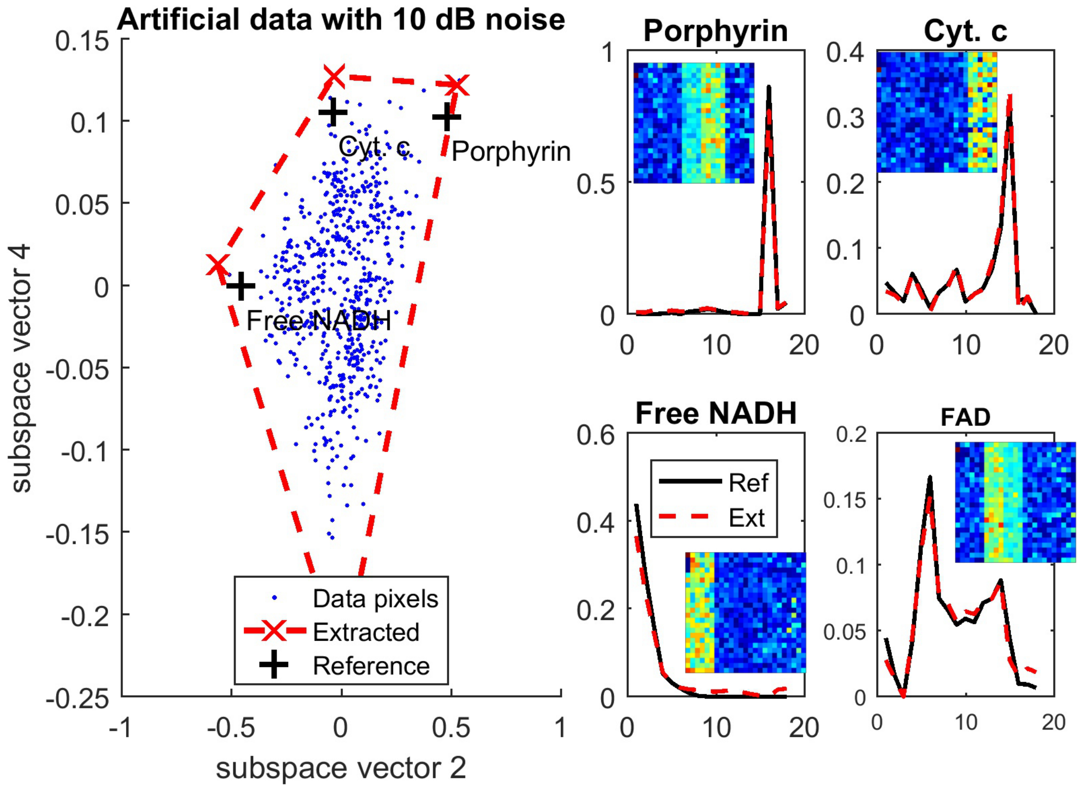

Figure 9: (clockwise)(first) Simplex representation of artificial data (Case B) with SNR of 10 dB. Blue dots represent 600 data points. Red crosses represent the extracted endmembers by unsupervised unmixing with red dotted enclosure. Reference spectra of free NADH, FAD, Cyt. C and Porphyrin are shown by black crosses. (second-fifth) Extracted and reference spectra marked with red colour and black colour respectively for free NADH, FAD, Cyt. C and Porphyrin. Here the spectra are presented with respect to spectral channels.

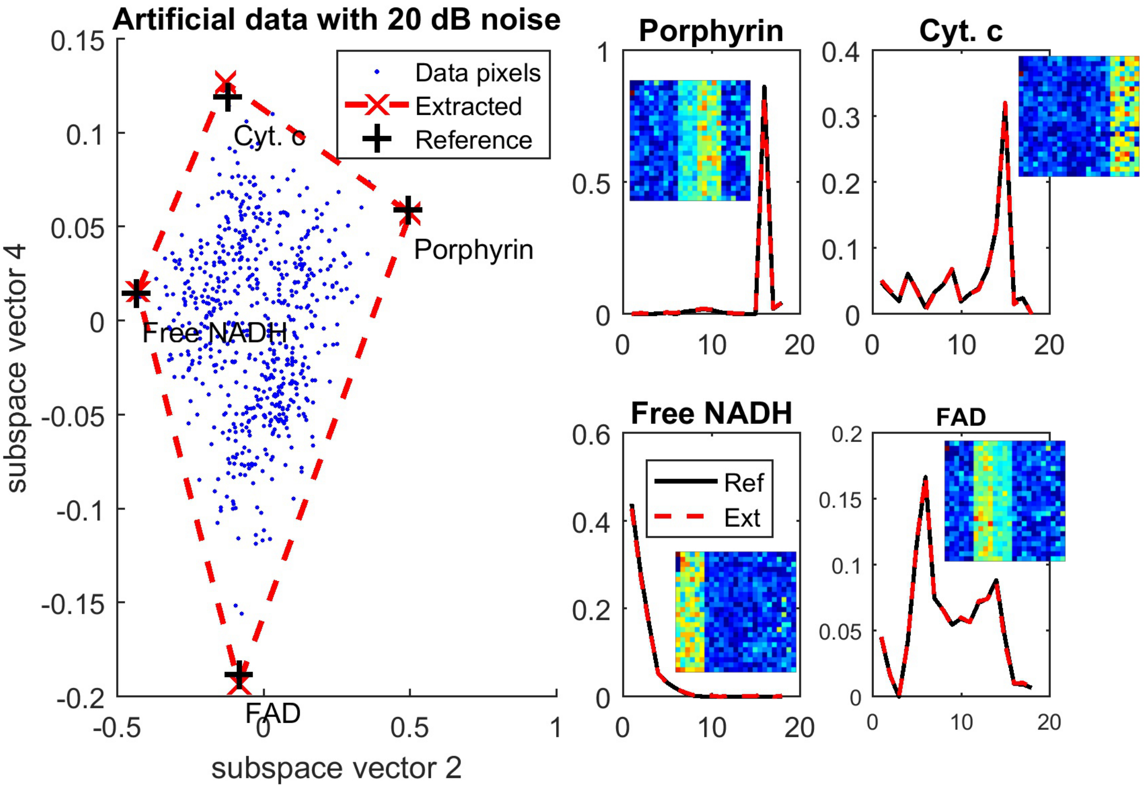

Figure 10: (clockwise)(first) Simplex representation of artificial data (Case B) with SNR of 20 dB. Blue dots represent 600 data points. Red crosses represent the extracted endmembers by unsupervised unmixing with red dotted enclosure. Reference spectra of free NADH, FAD, Cyt. C and Porphyrin are shown by black crosses. (second-fifth) Extracted and reference spectra marked with red colour and black colour respectively for free NADH, FAD, Cyt. C and Porphyrin. Here the spectra are presented with respect to spectral channels.

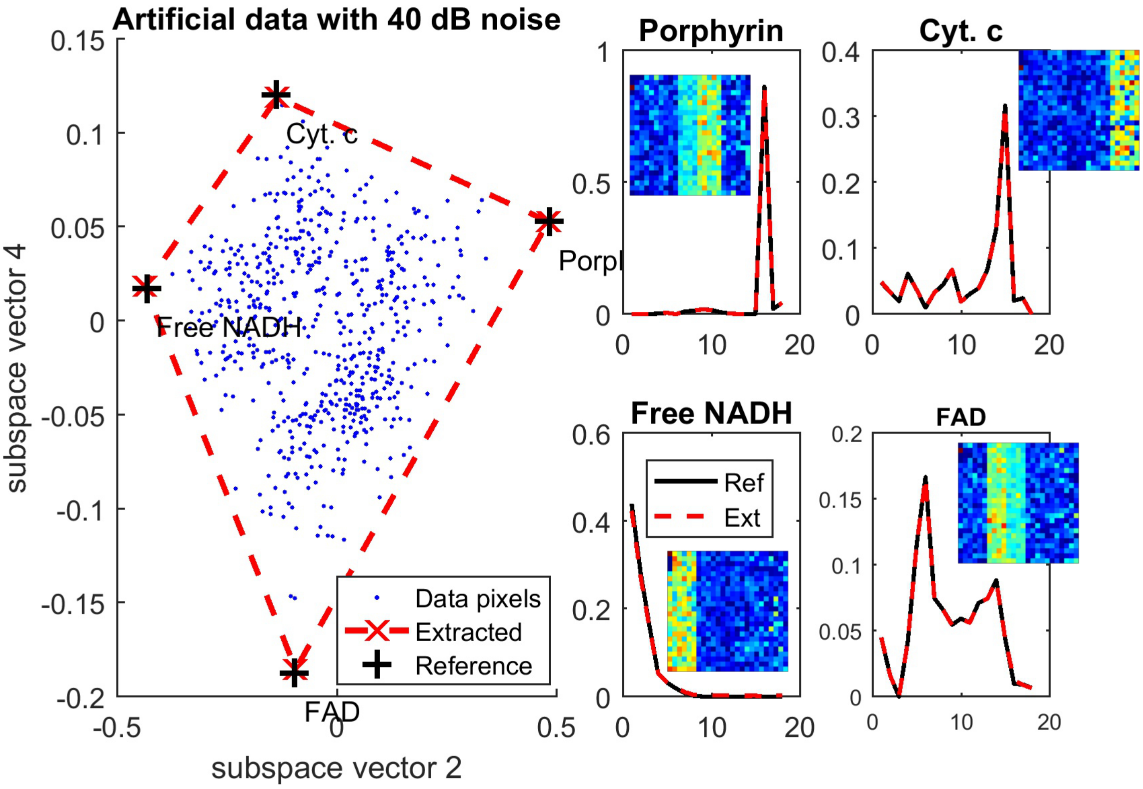

Figure 11: (clockwise)(first) Simplex representation of artificial data (Case B) with SNR of 40 dB. Blue dots represent 600 data points. Red crosses represent the extracted endmembers by unsupervised unmixing with red dotted enclosure. Reference spectra of free NADH, FAD, Cyt. C and Porphyrin are shown by black crosses. (second-fifth) Extracted and reference spectra marked with red colour and black colour respectively for free NADH, FAD, Cyt. C and Porphyrin. Here the spectra are presented with respect to spectral channels.

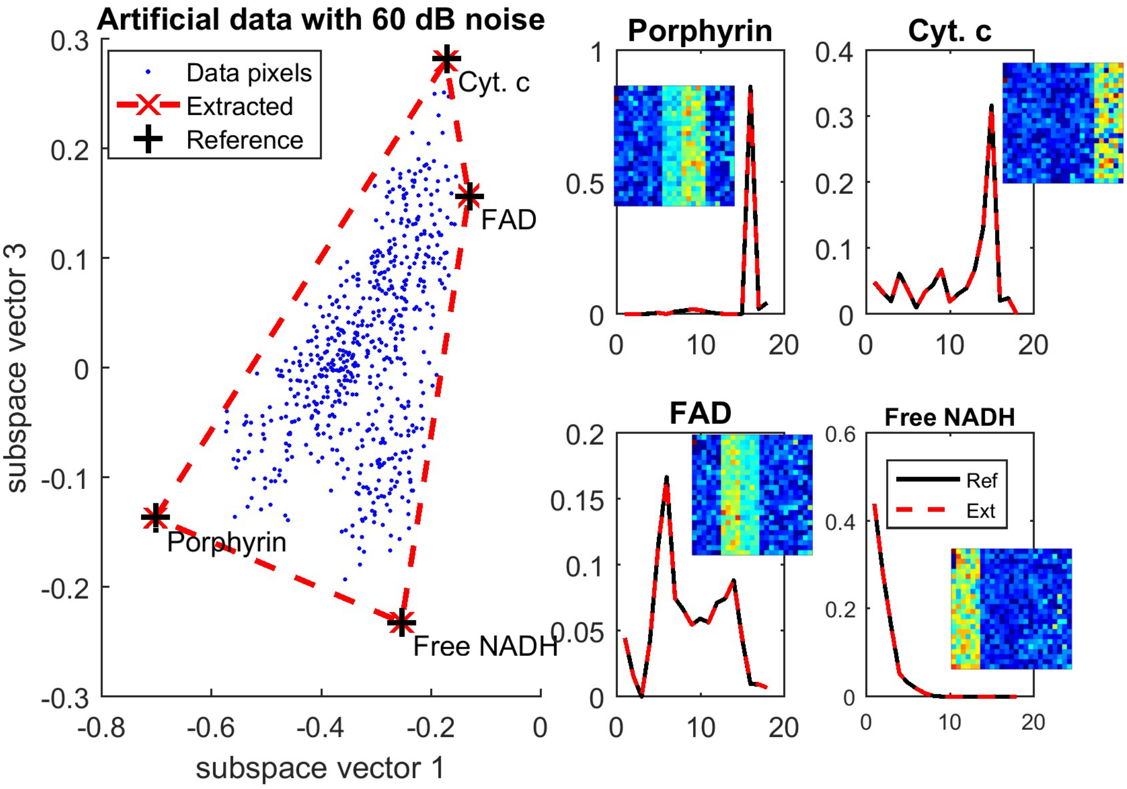

Figure 12: (clockwise)(first) Simplex representation of artificial data (Case B) with SNR of 60 dB. Blue dots represent 600 data points. Red crosses represent the extracted endmembers by unsupervised unmixing with red dotted enclosure. Reference spectra of free NADH, FAD, Cyt. C and Porphyrin are shown by black crosses. (second-fifth) Extracted and reference spectra marked with red colour and black colour respectively for free NADH, FAD, Cyt. C and Porphyrin. Here the spectra are presented with respect to spectral channels.

6.3 Artificial Data- Case C

We generated a pre-determined abundance map (Figure 13 (a-d)), by creating 4 two-dimensional images N=24 $\times$ 25 pixel representing maps of abundance fractions and assigned them to each of the four chosen fluorophores NADH, FAD, porphyrin and cytochrome complex, Cyt. C (Figure 13 e). In this case, FAD and porphyrin spatially overlap at column 11 to column 16 with the abundance fraction of around 50% for both FAD and porphyrin. Moreover, Free NADH and Cyt. c spatially overlap from row 11 to row 16 with the abundance fraction of around 50% for both Free NADH and Cyt. c.

In order to establish the robustness of RoDECA unmixing, this analysis was extended to cover a broad range of added Gaussian white noise with SNR from 10 dB to 60 dB. The simplexes of the artificial data as well as the spectra of extracted fluorophores compared with reference spectra for the SNR of 10 dB, 20 dB, 40 dB and 60 dB, are shown in Figures 14, 15, 16 and 17 respectively.

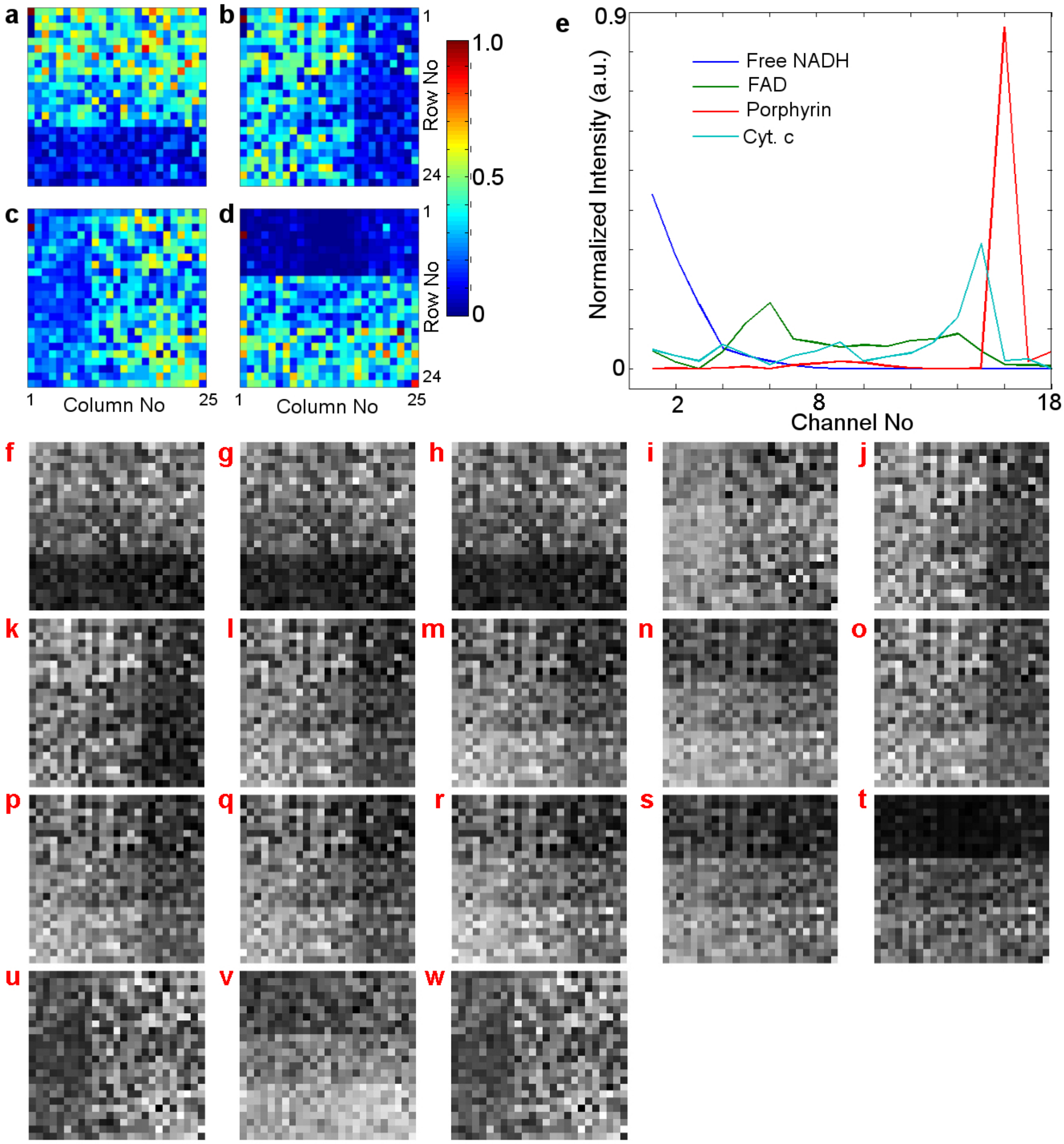

Figure 13: The artificial abundance map $\mathbf{s}_{[4\times600]}$ (for case C) of the total of 600 (24×25) pixels containing the fractions of each of four fluorophore (a) free NADH, (b) FAD, (c) porphyrin and (d) Cyt. C is presented. (e) The normalized reference spectra are shown for free NADH (blue), FAD (green), Cyt. C (cyan) and porphyrin (red). (f-w) Simulated hyperspectral image set (y=Ms+n) assuming zero noise (n=0) taken in L=18 different spectral channels. Images (f-w) are rescaled for better visualization as a grey colour-map, where the minimum value is displayed as black, and the maximum value (varying at each channel) is displayed as white.

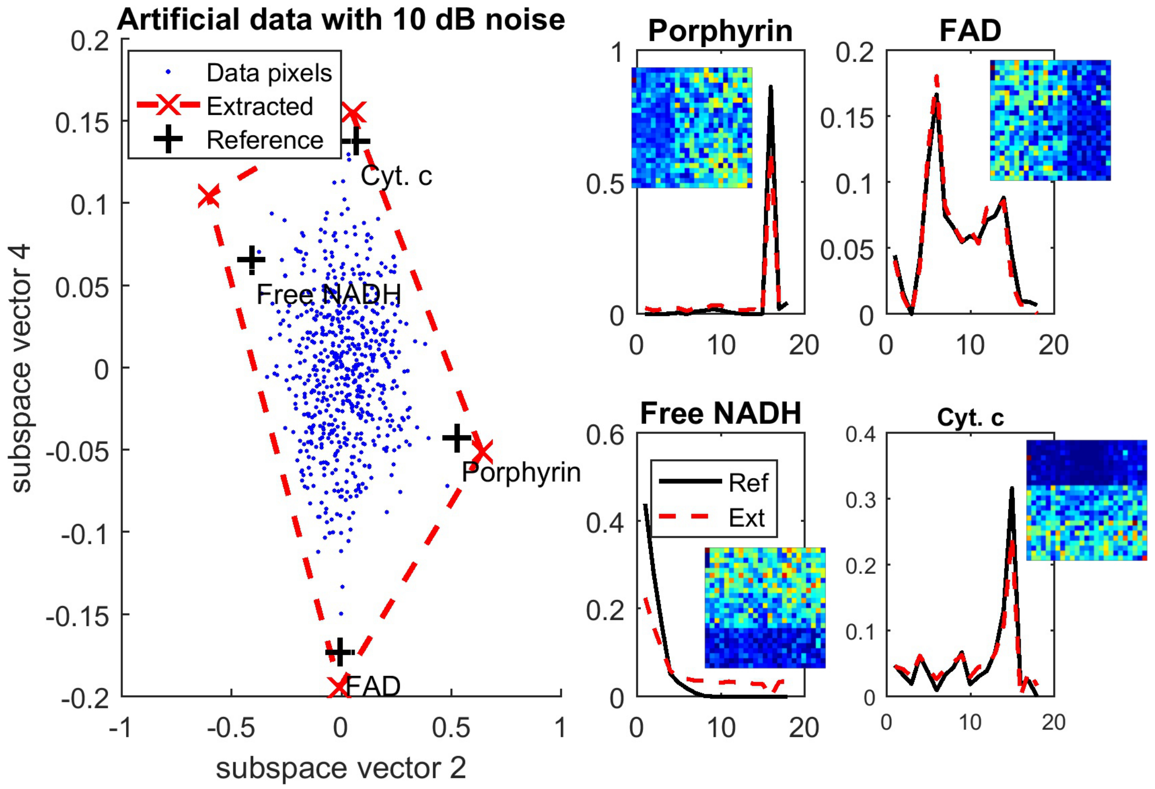

Figure 14: (clockwise)(first) Simplex representation of artificial data (Case C) with SNR of 10 dB. Blue dots represent 600 data points. Red crosses represent the extracted endmembers by unsupervised unmixing with red dotted enclosure. Reference spectra of free NADH, FAD, Cyt. C and Porphyrin are shown by black crosses. (second-fifth) Extracted and reference spectra marked with red colour and black colour respectively for free NADH, FAD, Cyt. C and Porphyrin. Here the spectra are presented with respect to spectral channels.

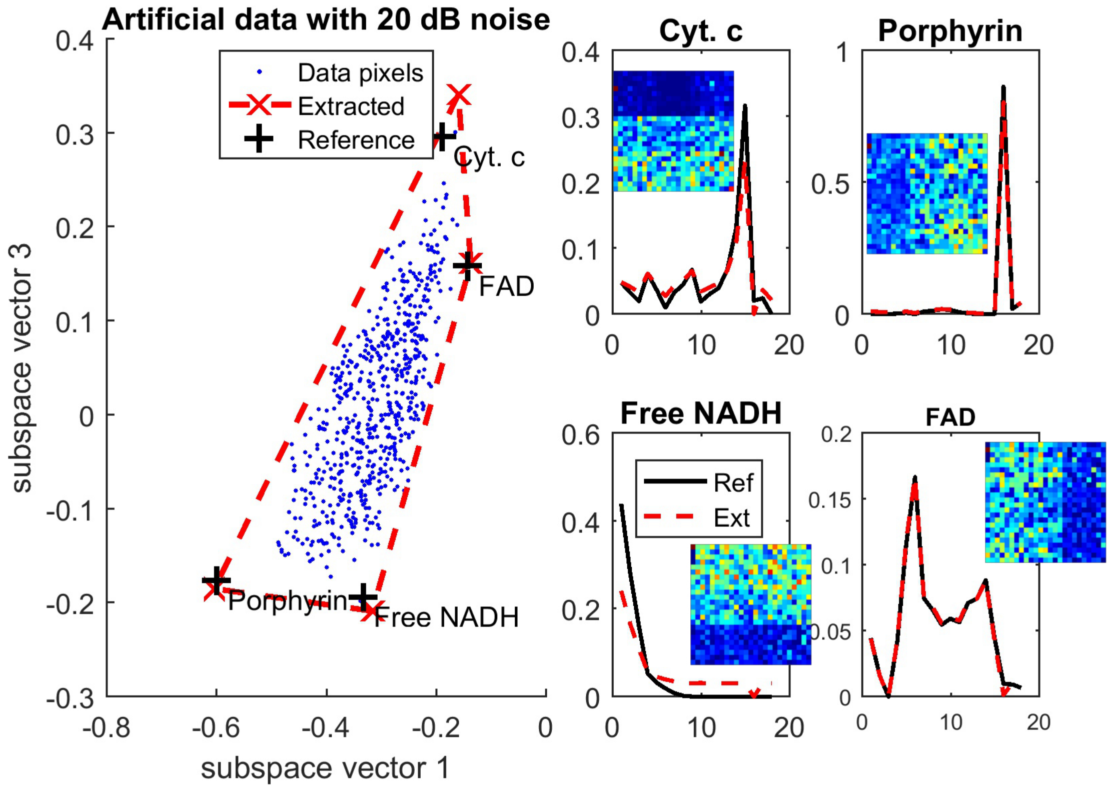

Figure 15: (clockwise)(first) Simplex representation of artificial data (Case C) with SNR of 20 dB. Blue dots represent 600 data points. Red crosses represent the extracted endmembers by unsupervised unmixing with red dotted enclosure. Reference spectra of free NADH, FAD, Cyt. C and Porphyrin are shown by black crosses. (second-fifth) Extracted and reference spectra marked with red colour and black colour respectively for free NADH, FAD, Cyt. C and Porphyrin. Here the spectra are presented with respect to spectral channels.

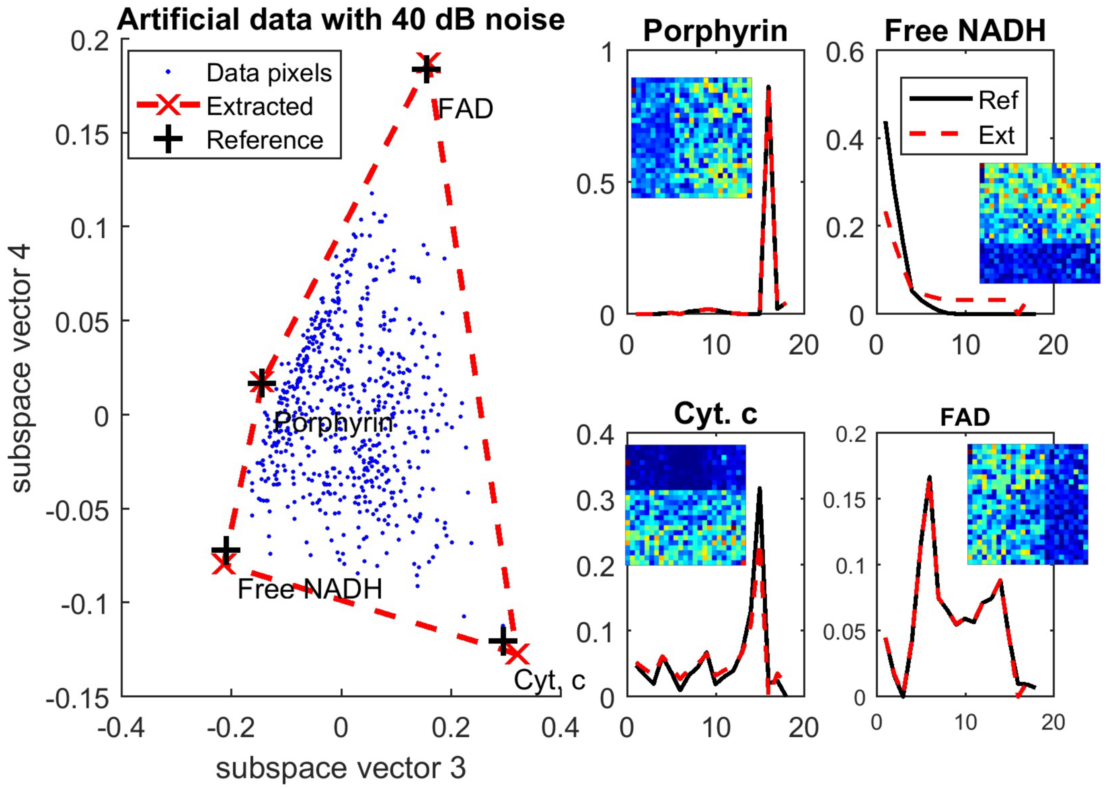

Figure 16: (clockwise)(first) Simplex representation of artificial data (Case C) with SNR of 40 dB. Blue dots represent 600 data points. Red crosses represent the extracted endmembers by unsupervised unmixing with red dotted enclosure. Reference spectra of free NADH, FAD, Cyt. C and Porphyrin are shown by black crosses. (second-fifth) Extracted and reference spectra marked with red colour and black colour respectively for free NADH, FAD, Cyt. C and Porphyrin. Here the spectra are presented with respect to spectral channels.

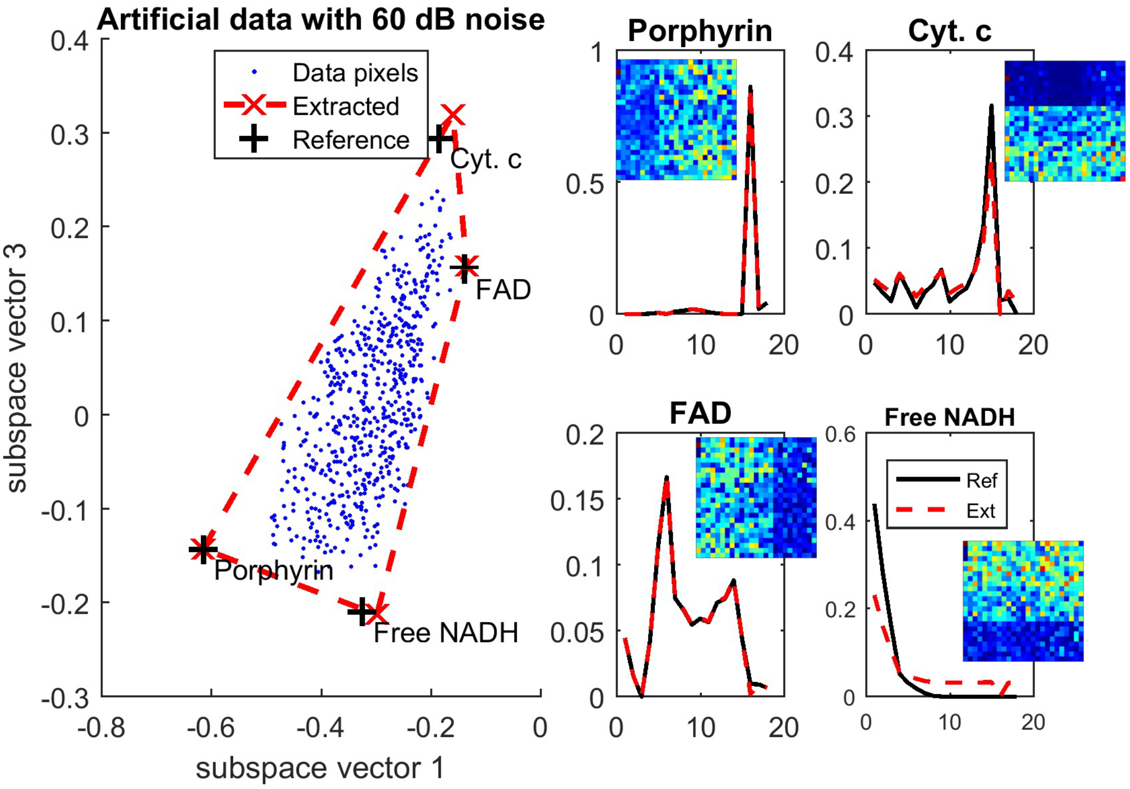

Figure 17: (clockwise)(first) Simplex representation of artificial data (Case C) with SNR of 60 dB. Blue dots represent 600 data points. Red crosses represent the extracted endmembers by unsupervised unmixing with red dotted enclosure. Reference spectra of free NADH, FAD, Cyt. C and Porphyrin are shown by black crosses. (second-fifth) Extracted and reference spectra marked with red colour and black colour respectively for free NADH, FAD, Cyt. C and Porphyrin. Here the spectra are presented with respect to spectral channels.

6.4 Artificial Data- Case D

we generated another dataset with a significant spectral overlap between two fluorophores and we chose two spectrally similar fluorophores, free NADH and bound NADH (main article in Figure 1 e). The generated abundance map (main article in Figure 1 (a-d))of two-dimensional images N=24×25 pixel representing maps of abundance fractions and assigned them to each of the four chosen fluorophores free NADH, bound NADH, porphyrin and cytochrome complex, Cyt. C (main article in Figure 1 e). In this case, bound NADH and porphyrin spatially overlap at column 11 to column 16 with the abundance fraction of around 50% for both bound NADH and porphyrin.

In order to establish the robustness of RoDECA unmixing, this analysis was extended to cover a broad range of added Gaussian white noise with SNR from 10 dB to 60 dB. The simplexes of the artificial data as well as the spectra of extracted fluorophores compared with reference spectra for the SNR of 10 dB, 40 dB , 20 dB and 10 dB are shown in Figures 18, 19, 20 and 21 respectively.

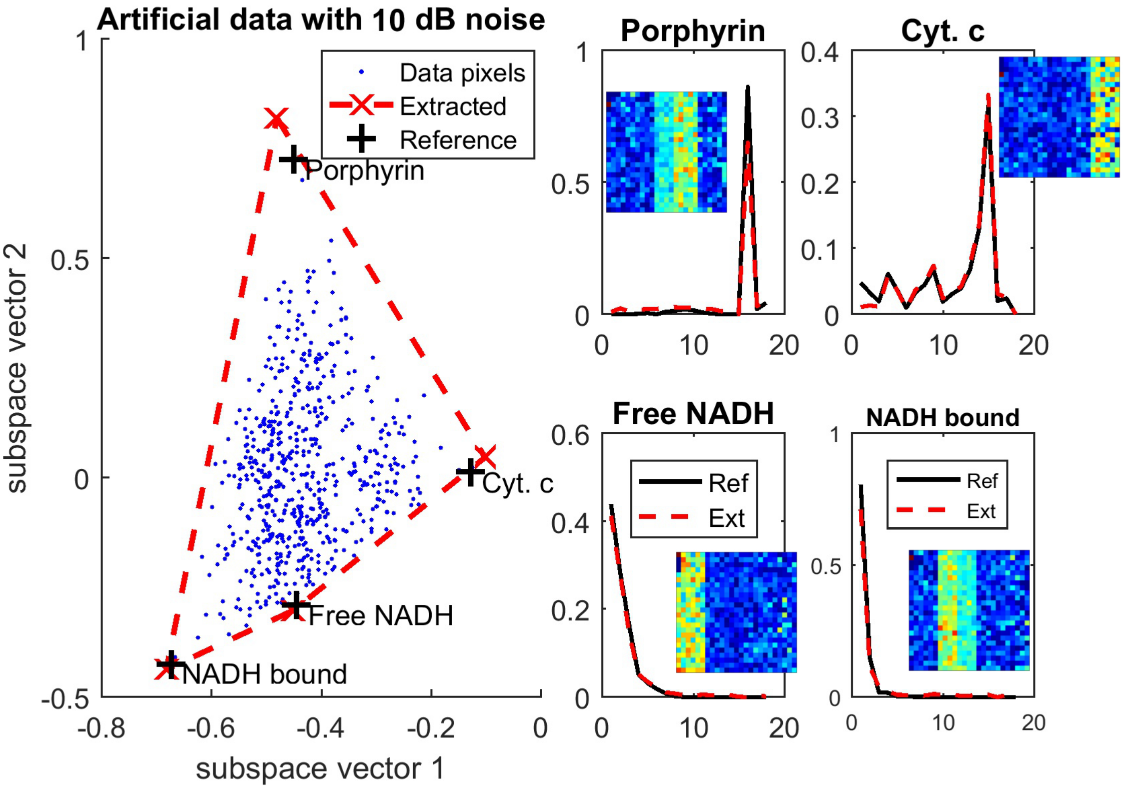

Figure 18: (clockwise)(first) Simplex representation of artificial data (Case D) with SNR of 10 dB. Blue dots represent 600 data points. Red crosses represent the extracted endmembers by unsupervised unmixing with red dotted enclosure. Reference spectra of free NADH, bound NADH, Cyt. C and Porphyrin are shown by black crosses. (second-fifth) Extracted and reference spectra marked with red colour and black colour respectively for free NADH, bound NADH, Cyt. C and Porphyrin. Here the spectra are presented with respect to spectral channels.

The root mean square error of abundance $\xi_{(s)}$ was determined by using Equation 7 (in main paper) and the spectrum error ξ_M was calculated by using Equation 8 (in main paper).

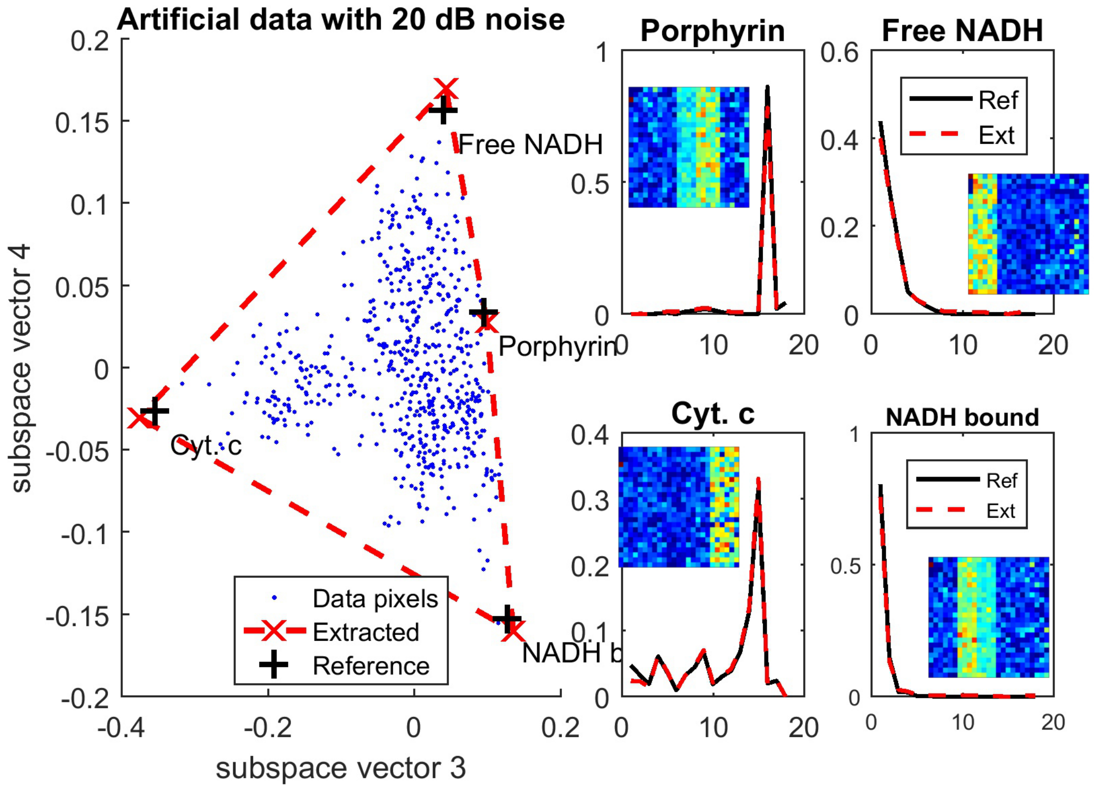

Figure 19: (clockwise)(first) Simplex representation of artificial data (Case D) with SNR of 20 dB. Blue dots represent 600 data points. Red crosses represent the extracted endmembers by unsupervised unmixing with red dotted enclosure. Reference spectra of free NADH, b-NADH, Cyt. C and Porphyrin are shown by black crosses. (second-fifth) Extracted and reference spectra marked with red colour and black colour respectively for free NADH, b-NADH, Cyt. C and Porphyrin. Here the spectra are presented with respect to spectral channels.

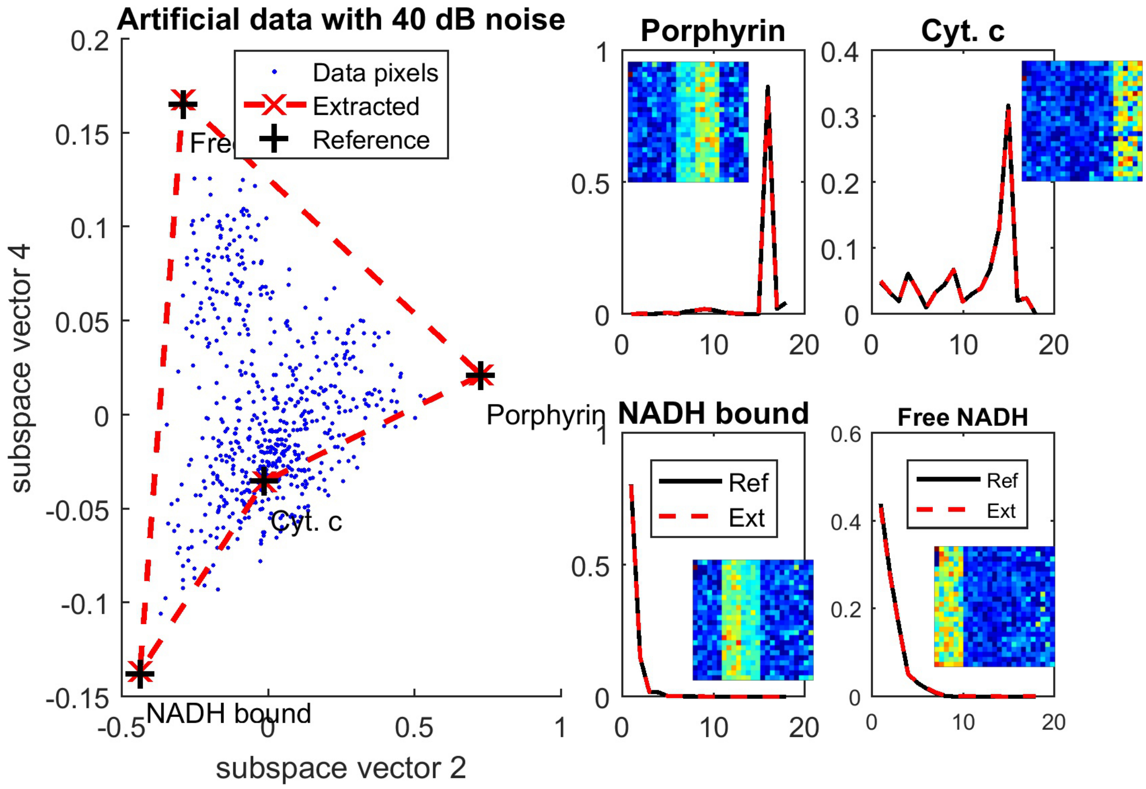

Figure 20: (clockwise)(first) Simplex representation of artificial data (Case D) with SNR of 40 dB. Blue dots represent 600 data points. Red crosses represent the extracted endmembers by unsupervised unmixing with red dotted enclosure. Reference spectra of free NADH, b-NADH, Cyt. C and Porphyrin are shown by black crosses. (second-fifth) Extracted and reference spectra marked with red colour and black colour respectively for free NADH, b-NADH, Cyt. C and Porphyrin. Here the spectra are presented with respect to spectral channels.

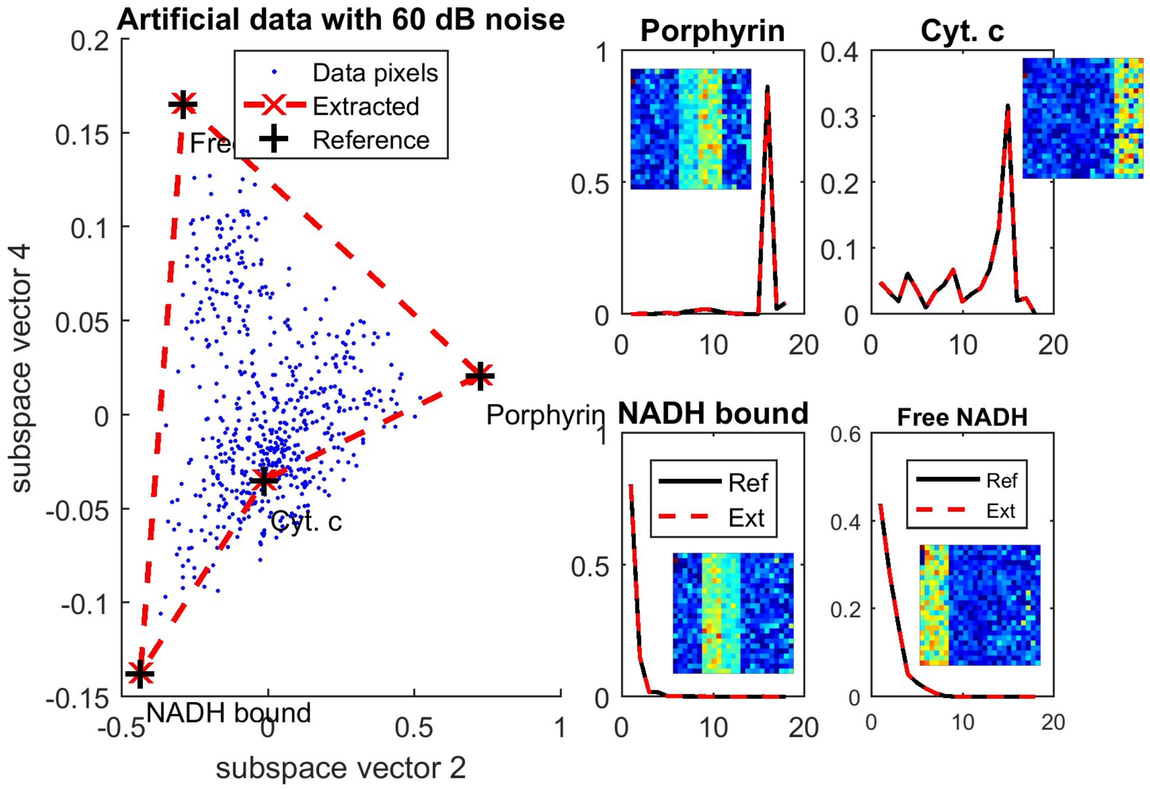

Figure 21: (clockwise)(first) Simplex representation of artificial data (Case D) with SNR of 60 dB. Blue dots represent 600 data points. Red crosses represent the extracted endmembers by unsupervised unmixing with red dotted enclosure. Reference spectra of free NADH, b-NADH, Cyt. C and Porphyrin are shown by black crosses. (second-fifth) Extracted and reference spectra marked with red colour and black colour respectively for free NADH, b-NADH, Cyt. C and Porphyrin. Here the spectra are presented with respect to spectral channels.

The result of these added Gaussian white noise with SNR from 10 dB to 60 dB is shown in Figure 22 and 23 respectively.

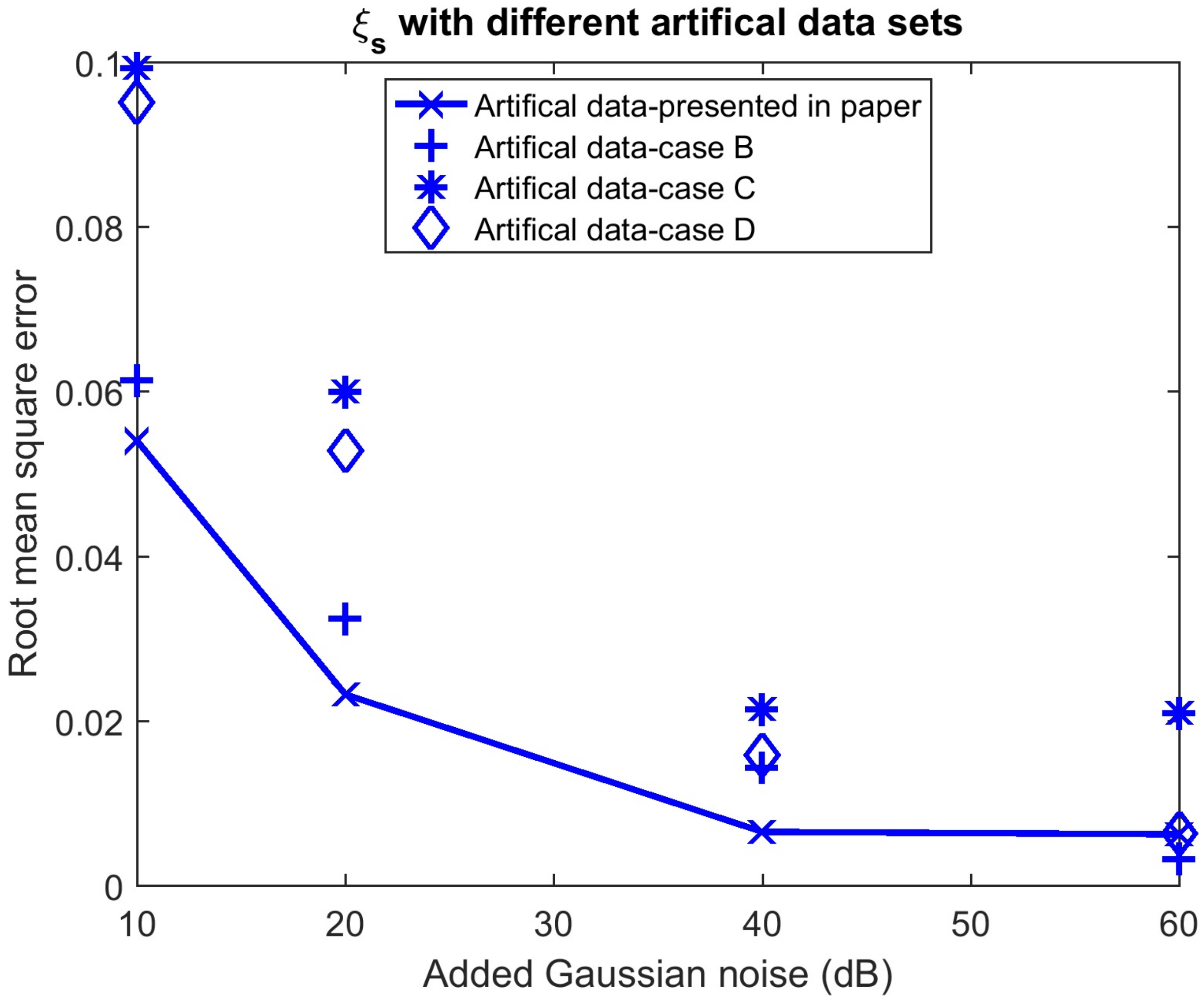

Figure 22: Root mean square error calculations of the extracted abundance matrices with various levels of applied Gaussian noise $\mathbf{n}$ for different cases of artificial data.

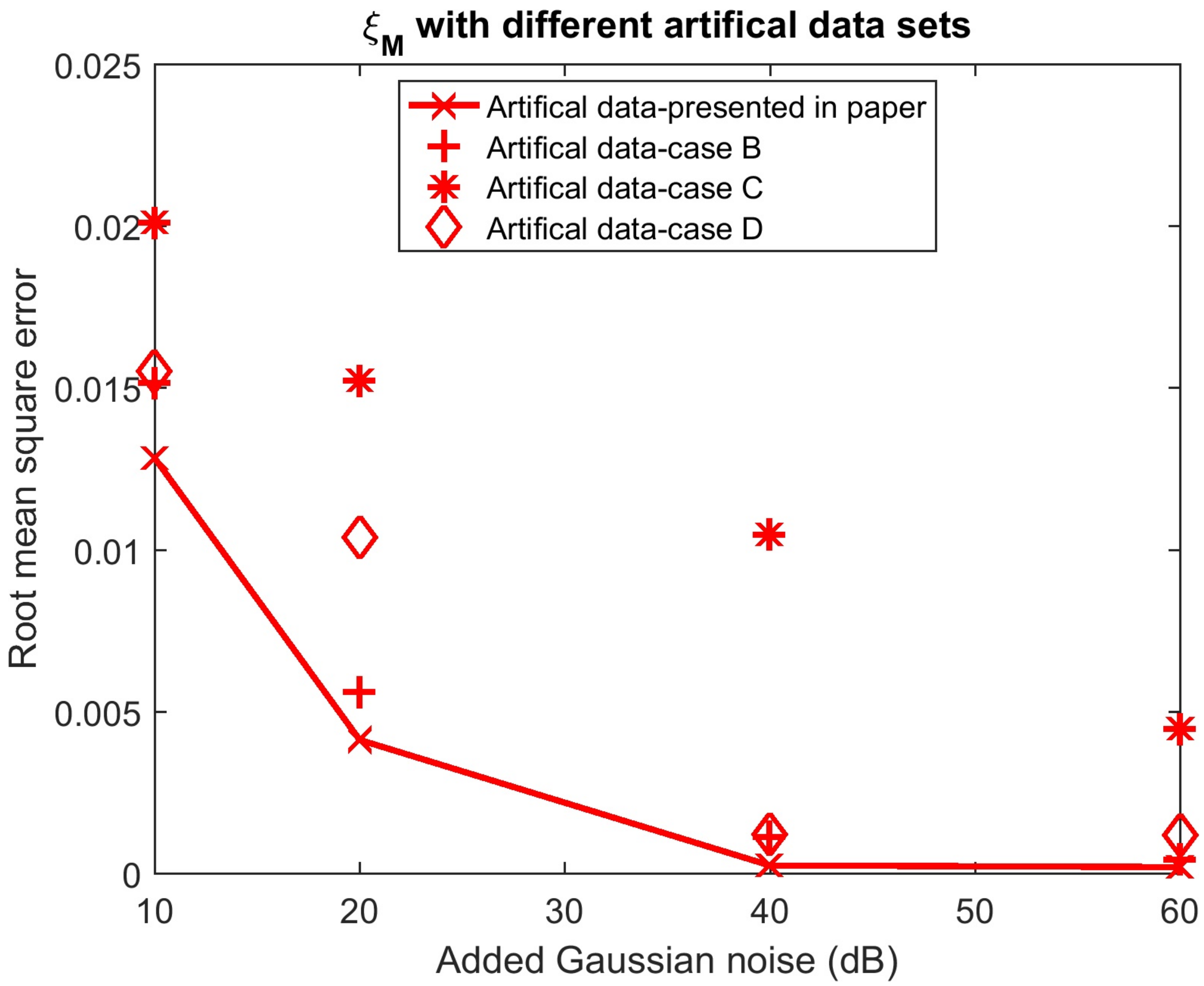

Figure 23: Root mean square error calculations of the extracted spectrum matrices with various levels of applied Gaussian noise $\mathbf{n}$ for different cases of artificial data.

6.5 Benchmarking with other methods

We performed the additional study to benchmark RoDECA with other well-established methods including SISAL, VCA, DUSAL and MVSA. The results are shown in Supplementary Figure 24. The root mean square error of spectrum $\xi_{(M)}$ of RoDECA is 0.0165, while it is 0.0867, 0.0566, 0.0897 and 0.0667 for SISAL, VCA, MVSA, and DUSAL respectively.

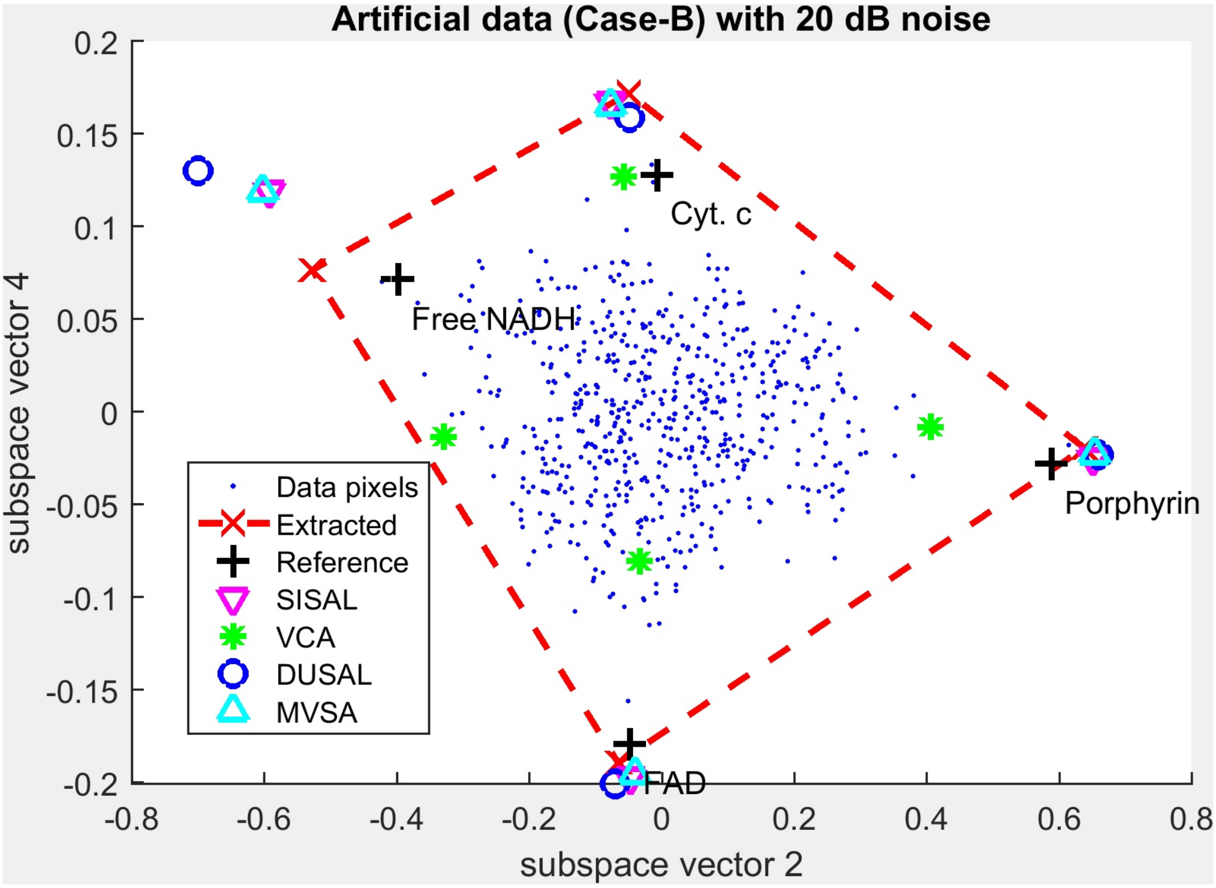

Figure 24: Simplex representation of artificial data-case B with SNR of 20 dB. Blue dots represent 600 image data points in our artificial data, while red crosses represent the extracted endmembers by the RoDECA method. Red dotted line joins the red crosses. The reference spectra of free NADH, b-NADH, Cyt. C and Porphyrin are shown by black crosses. The endmembers estimated using these methods are indicated as follows: SISAL (magenta inverted triangles), VCA (green asterisks), DUSAL (blue circles), MVSA (cyan upright triangles).

<u>List of the symbols</u>

<u>(In chronological order from the main Article)</u>

- N = total pixels each.
- L = different spectral channels (L=18 in our case study).
- p = Number of estimated or extracted endmembers through HySime with respect to unsupervised method. Normally the estimated endmembers are lower than the total number of the spectral channels ($p < L$).
- i = pixel operator ranging from 1,…, N.
- k = channel operator ranging from 1,…, L.
- j = extracted endmember's operator ranging from 1,…, p.
- $\boldsymbol{y} = [\, y_{ki}\,]$ = The recorded fluorescence signals from the hyperspectral dataset termed by a matrix where y_{ki} is the pixel value in channel k, (k=1,..,L) of image pixel i, (i=1,...,N).
- $\boldsymbol{M} = [M_{kj}]$ = Endmember matrix (where, k=1,...,L, j=1,...,p).
- $\boldsymbol{s} = [s_{ji}]$, (j=1,...,p , i=1,...,N) = abundance fractions specified in the abundance matrix.
- $\boldsymbol{x} = [x_{ki}]$ = The recorded fluorescence signals form the hyperspectral dataset termed by a matrix where x_{ki} is the pixel value in channel k, (k=1,..,L) of image pixel i, (i=1,...,N) <u>without any noise</u>. The relationship between $\boldsymbol{x}$ and $\boldsymbol{y}$ is denoted simply by adding image noise ($\boldsymbol{n}$) and the signal will be; $\boldsymbol{y} = \boldsymbol{x} + \boldsymbol{n}$.
- $\boldsymbol{n}$ = image noise.
- $\overrightarrow{y_i}$ = vector forming columns of the matrix $\boldsymbol{y}$ **,** represent the pixel spectra at L-dimensional space.
- $\overrightarrow{M_j}$ = vector forming columns of matrix $\boldsymbol{M}$ represent the endmember spectra at p dimensional space.
- $\boldsymbol{E}_p$ = rotational matrix for optimum subspace. The subset which best satisfies this criterion is then selected as the set of basis vectors that is $\boldsymbol{E}_p$ for the optimised signal subspace.

Figure 25: list of symbols 1 of 2

- y_{pi} = Input signal data at reduced p subspace $\mathrm{w.r.t.}\, \boldsymbol{E}_p$, $\mathbb{R}^{p\times p}$.
- $\boldsymbol{A}$ = Intermediate endmember matrix. In DECA analysis, it estimates the intermediate endmember matrix $\boldsymbol{A}$ ($\boldsymbol{A} \in p\ subspace\ w.r.t.\boldsymbol{E}_p$, $\mathbb{R}^{p\times p}$) blindly by modelling the abundance matrix $\boldsymbol{s}$ as a mixture of Dirichlet densities.
- $\boldsymbol{W} \equiv\ \boldsymbol{A}^{-1}$ = Intermediate endmember matrix for subtracting the abundance map $\boldsymbol{s}$ at p subspace.
- $y_{k,i\ [digital]}$ = Denotes the arbitrary unit in digital counts with EMCCD camera for the imaged sample.
- $BO_{[digital]})$ = Bias Offset.
- se= sensitivity
- G_{EM} = EM gain
- QE = Quantum efficiency of camera sensor based on wavelength
- t_{expo} = Exposure time of the EMCCD camera.
- $B(k,i)$ = The average of two smoothed background images.
- $C_{raw}(k,i)$ = The average value of smoothed calibration fluid.
- $f(k)$ = The normalized spectrum of the calibration fluid with respect to channels. This 1-D value is used to correlate the hyperspectral images with fluorescence spectra.
- ξ_M = Spectra error which is calculated at a channel level w.r.t $M_{k,cal}$ and $M_{k,org}$
- $M_{k,cal}$ = Extracted/ calculated mixing matrix by modified DECA.
- $M_{k,org}$= Original mixing matrix for the artificial dataset.
- ξ_s = Pixel wise abundance error with respect to $s_{cal}(j,i)$ and $s_{org}\,(j,i)$.
- $s_{cal}(j,i)$= Extracted/ calculated abundance matrix by modified DECA.
- $s_{org}\,(j,i)$ = Original abundance matrix for the artificial dataset.

Figure 26: list of symbols 2 of 2

In chronological order from the supplementary material

(except duplicate symbols from earlier list)

- $\mathbb{R}^L$ = matrix with real number at L-dimension
- $\mathbf{E} \equiv [\mathbf{e}_1, \ldots, \mathbf{e}_L]$ = matrix of eigenvectors
- $\mathbf{U}_k$ = projection matrix
- $\mathbb{E}[\widehat{\mathbf{x}}_k|\mathbf{x}]$ = conditional expected mean value of the projected vector $\widehat{\mathbf{x}}_k$ given condition $\mathbf{x}$
- $\widehat{\mathbf{x}}_k$ = projected signal of $\mathbf{x}_k$
- $\mathbf{R_n}$ = noise correlation matrix
- $D\left(\mathbf{s} \mid \theta_{\mathbf{q}}\right)$ = Dirichlet density of $\mathbf{s}$ with the parameter $\boldsymbol{\theta}_q$
- $\mathcal{L}\left(\mathbf{W}, \boldsymbol{\theta}\right)$ = log-likelihood of $\mathbf{W}$ with the set of the parameters $\boldsymbol{\theta}$
- $\mathcal{X}$ = incomplete data
- $\mathcal{Z}$ = missing data
- $Q\left(\boldsymbol{\theta}, \mathbf{W};\ \widehat{\boldsymbol{\theta}}^{(t)}, \widehat{\mathbf{W}}^{(t)}\right)$ = Q-function is used in EM algorithm
- $\mathbf{M_{int}}$ = intermediate matrix of endmember spectra
- $\mathbf{M_{int}}'$ = rescaled matrix of endmember spectra
- $\mathbf{M_{int}}''$ = normalized-positive matrix of endmember spectra

References

[1] Ahmed R Abas. Unsupervised learning of mixture models based on swarm intelligence and neural networks with optimal completion using incomplete data. *Egyptian Informatics Journal*, 13(2):103–109, 2012.

[2] Manya V Afonso, José M Bioucas-Dias, and Mário AT Figueiredo. Fast image recovery using variable splitting and constrained optimization. *IEEE Transactions on Image Processing*, 19(9):2345–2356, 2010.

[3] José M Bioucas-Dias. A variable splitting augmented lagrangian approach to linear spectral unmixing. In *2009 First Workshop on Hyperspectral Image and Signal Processing: Evolution in Remote Sensing*, pages 1–4. IEEE, 2009.

[4] Patrick L Combettes and Valérie R Wajs. Signal recovery by proximal forward-backward splitting. *Multiscale Modeling & Simulation*, 4(4):1168–1200, 2005.

[5] Mario A. T. Figueiredo and Anil K. Jain. Unsupervised learning of finite mixture models. *IEEE Transactions on pattern analysis and machine intelligence*, 24(3):381–396, 2002.

[6] Tom Goldstein and Stanley Osher. The split bregman method for l1-regularized problems. *SIAM journal on imaging sciences*, 2(2):323–343, 2009.

[7] Wolfgang Gross, Hendrik Schilling, and Wolfgang Middelmann. An approach to fully unsupervised hyperspectral unmixing. In *2012 IEEE International Geoscience and Remote Sensing Symposium*, pages 4714–4717. IEEE, 2012.

[8] K Lange. Optimization springer texts in statistics. 2004.

[9] Geoffrey McLachlan and Thriyambakam Krishnan. *The EM algorithm and extensions*, volume 382. John Wiley & Sons, 2007.

[10] Geoffrey McLachlan and David Peel. *Finite mixture models*. John Wiley & Sons, 2004.

[11] Thomas Minka. Estimating a dirichlet distribution, 2000.

[12] Jose Manuel Peixoto Nascimento. *Unsupervised hyperspectral unmixing*. PhD thesis, Universidade Técnica de Lisboa, 2006.

[13] José MP Nascimento and José M Bioucas-Dias. Hyperspectral signal subspace estimation. In *2007 IEEE International Geoscience and Remote Sensing Symposium*, pages 3225–3228. IEEE, 2007.

[14] José MP Nascimento and José M Bioucas-Dias. Hyperspectral unmixing based on mixtures of dirichlet components. *IEEE Transactions on Geoscience and Remote Sensing*, 50(3):863–878, 2012.
